# Feasibility and immediate effects of a multimodal, dance-integrated educational video package on eye health knowledge and intentions in Nigeria: A pre-post pilot study

**DOI:** 10.1371/journal.pgph.0005585

**Published:** 2026-10-14

**Authors:** Ngozi Precious Uwagboe, Adaku Israel Obia, Onyinye Lilian Nwaolai, Pemije Gadimoh, Eghosa Osahon Alari, Carolyne Naana Yanney, Nnamdi John Eseme, Uchechukwu Levi Osuagwu

**Affiliations:** 1 Department of Optometry and Vision Sciences, Faculty of Optometry, University of Benin, Benin City, Nigeria; 2 Magnificent Eyecare Centre Limited, Federal Capital City, Abuja, Nigeria; 3 Department of Animal and Environmental Biology, Laboratory of Parasitology Research, University of Benin, Benin City, Nigeria; 4 Department of Optometry and Visual Science, Kwame Nkrumah University of Science and Technology, Kumasi, Ghana; 5 Menzies Institute for Medical Research, University of Tasmania, Hobart, Tasmania, Australia; 6 Bathurst Rural Clinical School (BRCS), School of Medicine, Faculty of Health, Western Sydney University, Bathurst, New South Wales, Australia; 7 Centre for Eye and Public Health Intervention Initiative (CEPHII), African Vision Research Institute, Discipline of Optometry, University of KwaZulu-Natal, Durban, South Africa; University of St Andrews, UNITED KINGDOM OF GREAT BRITAIN AND NORTHERN IRELAND

## Abstract

Dance, as a culturally embedded and universally accessible form of art, offers a promising medium for health education in settings with diverse literacy levels. This pilot study evaluated the feasibility and immediate effects of a multimodal, dance-integrated educational video package designed to improve eye health knowledge, attitudes, and preventive intentions among Nigerian adults. Between April 11 and June 22, 2025, adults recruited through optometry clinics and community outreach programmes viewed culturally tailored educational videos addressing at least one eye condition (myopia, cataract, or glaucoma) and general eye health, including common myths and preventive practices. Pre–post changes were assessed using paired comparisons, with subgroup analyses by educational attainment. Among 438 participants, 77.7% were aged 18–44 years, 52.5% were female, 75.1% had higher education, 80.0% reported existing health-promoting behaviours, and 79.7% considered regular eye examinations important. Following the intervention, perceived eye health knowledge increased from 42.0% to 68.0% (absolute increase, 26.0 percentage points; relative increase, 61.9%; p = 0.013; Cramér’s V = 0.279), while objective eye health knowledge scores increased by 52.4 percentage points (p = 0.013; Cramér’s V = 0.069). Self-reported intentions to adopt protective practices, including wearing sunglasses, reducing screen time, and attending regular eye examinations, also increased. Overall intention to visit an eye clinic increased by 10%, although this change was not statistically significant (p = 0.443), likely reflecting high baseline health-seeking intentions. The largest improvements in willingness to attend regular check-ups occurred among participants with secondary education (27.5% to 96.3%, p < 0.001) and those with primary or no formal education (41.4% to 96.6%, p < 0.001). Dance-integrated educational videos were associated with immediate improvements in eye health knowledge and preventive intentions, particularly among participants with lower educational attainment, supporting their potential as a culturally relevant and scalable approach to eye health communication in diverse, resource-limited settings and community-based health promotion programmes.

## Introduction

Visual impairment and blindness remain pressing global public health challenges, with refractive errors and cataracts being the leading causes of these conditions [1]. Vision impairment has been defined as a limitation of one or more functions of the eye or visual system, including a reduced ability to see clearly (visual acuity) or a limited range of vision (visual field) that cannot be fully corrected by conventional means such as spectacles or contact lenses [2]. The severity of visual impairment might range from mild to complete blindness, and is determined by assessing the best corrected visual acuity in the better eye [2,3]. Globally, an estimated 2.2 billion people are affected by some form of vision impairment, and for at least 1 billion of these individuals, their condition could have been prevented or remains untreated due to a lack of timely intervention and awareness [1,4].

This global burden is disproportionately concentrated in low- and middle-income countries (LMICs), including Nigeria, where systemic and cultural barriers persist, such as limited access to eye care, cultural misconceptions, and poor literacy hinder prevention and early treatment [5–7]. For example, in Nigeria, more than 4.25 million people are affected by preventable blindness or moderate-to-severe visual impairment, primarily from avoidable causes [8–10]. Despite the availability of sight-restoring surgery, uptake within Nigeria remains low [11], largely due to poor eye health literacy and ocular myths, such as the belief that spectacles weaken vision or that herbal remedies can cure eye conditions [12–15].

Health literacy has been defined as the achievement of a level of knowledge, skills, and confidence to take action to improve one’s health by changing lifestyles and living conditions [16]. Researchers have argued that, as a social determinant of health, health literacy can be achieved through health education or health promotion [17–19]. However, it should be noted that the constructs of health education and health promotion are related, but distinct [20–25]. In this study, the health promotion component is embodied in the use of culturally engaging dance-integrated videos intended to encourage behaviour change, while the health education component is expressed in the supply of correct information about myopia, cataract, glaucoma, and ocular myths. Specifically, the dance component functions as a primary health promotion mechanism by fostering emotional engagement, leveraging cultural resonance through familiar rhythms, and providing social modelling; these elements work synergistically to reduce psychological barriers and motivate behavioural change beyond the mere delivery of factual information. By integrating these concepts, the dance-integrated educational videos, not only increases awareness but also boosts participation and promotes the adoption of preventive eye health behaviours.

Therefore, improving eye health literacy through effective health education and promotion strategies is critical for preventing avoidable visual impairment and blindness. Over the years, traditional health promotion tools, including posters, leaflets, and radio broadcasts, have been used to drive health literacy in Nigeria; however, this approach has achieved only modest success, especially in low-literacy communities [26,27]. Evidence from other health domains suggests that culturally tailored, participatory education approaches can significantly improve health literacy and encourage earlier care-seeking behaviour [28–31]. Among these participatory methods, performing arts offer a unique avenue for engaging communities through entertainment and cultural expression. Research shows that dance, a deeply embedded cultural practice in Nigeria and many African countries, offers benefits in merging health literacy with entertainment in community health promotion interventions [32–36]. Historically, performing arts such as travelling theatre and community-based dance have served as powerful conduits for social change and public health communication in Nigeria [37]. These traditional modalities are deeply rooted in the country’s communal and oral traditions and have historically served as effective conduits for disseminating health information within Nigerian communities. While such communal, oral art forms are often credited with reaching audiences regardless of literacy level, participants in the present study were required to be literate in English, so this particular rationale does not directly apply here. Rather, the suitability of dance and allied performing arts for eye health promotion lies primarily in their capacity to sustain audience engagement: lengthy, text-based health information can be easy to disengage from, whereas interactive, dance-based videos with text overlays may capture attention and encourage fuller reading and retention of the information provided. Dance has accordingly been leveraged in health campaigns to attract attention, enhance recall, and foster community-level diffusion of health messages [32–36,45,46]. In Ethiopia, the use of cultural dances enhanced physiological parameters among adolescents [35], while culturally rooted dance activities improved cardiovascular health and self-efficacy in older Mozambican women [33]. However, the use of dance and allied performing arts remains an underutilised resource for eye health education and promotion, especially in low-literacy and resource-constrained settings.

This study evaluated the effectiveness of dance-integrated educational videos in improving awareness, attitudes, and preventive practices related to myopia, cataract, glaucoma, and ocular myths among adult Nigerians.

## Methods

### Study design

This was a quantitative pre- and post-intervention educational exposure study conducted from April 11 to June 22, 2025. The structured pre- and post-intervention design allowed for the immediate assessment of the intervention’s impact by collecting data at a single time point, thereby avoiding the need for longitudinal follow-up [38]. The reporting of this study adheres to the principles of the TREND (Transparent Reporting of Evaluations with Non-randomised Designs) statement for quasi-experimental designs [39]. see Supplementary File S1 Table.

### Recruitment

Participating clinics were identified and invited to indicate their interest in supporting the Dance-Integrated Video Educational Project prior to participant recruitment. A list of clinics that had expressed interest was established across the relevant geographical zones.Participant recruitment was conducted during pre-scheduled community outreach activities undertaken by the student researchers in these zones. During the outreaches, researchers provided community members with information about the study and invited those who were interested to participate. Individuals who expressed interest were informed that they could attend any of the participating clinics in their area at a convenient time to complete the study procedures.The participating clinics served primarily as designated meeting points for study participation and data collection, rather than as the primary source of recruitment. In Nigeria, recruiting solely from clinical lists would introduce profound selection bias and exclude the most vulnerable populations who face geographic and financial barriers to care. Using community outreaches as the primary recruitment vehicle, while designating local clinics as central, safe, and equipped meeting hubs, is an established and highly pragmatic strategy to bridge the gap in care.

At the clinic visit, a researcher assigned to the respective clinic explained the study to the participant, provided the patient information sheet, and obtained written informed consent before administering the study questionnaire. No financial or other incentives were provided to participants for taking part in the study. Participation was voluntary, and individuals were free to decline or withdraw from the study without consequence. Participating clinics (both public and private) across Nigeria’s six geopolitical zones were purposively selected as meeting points for participants.

Eligible clinics were required to have a quiet environment, adequate seating, and functional audio-visual equipment for video presentation. Only clinics providing institutional consent and meeting these criteria participated in the study.

Participants were Nigerian adults aged 18 years or older recruited to the participating clinics. Inclusion criteria for participants included the ability to read and write in English and the provision of a written informed consent. Individuals with significant cognitive or hearing impairments that could interfere with participation in the study were excluded.

### Sample size determination

Sample size was calculated using Cochran’s formula for unknown population proportions: $n=\frac{{\text{Z}}^{\text{2}}\cdot \text{P}(\text{1}-\text{P})}{{\text{d}}^{\text{2}}}$, where *Z* = 1.96 (95% confidence level), *P* = 0.50 (maximum variability), and *d* = 0.05 (margin of error), yielding *n* = 385*.* Factoring in a 10% non-response rate *(n* = 39*),* the final target sample size was established at *N* = 424 participants*.*

### Intervention

The intervention consisted of four culturally tailored, dance-integrated educational videos on eye health, each lasting approximately three minutes (see Videos: 10.6084/m9.figshare.33227856). The individual links to the video are provided as supplementary Files S1 File (S1 Video, S2 Video, S3 Video, and S4 Video) for myopia, Cataract, Glaucoma, and dispelling myths about ocular problems, respectively. As this was an exploratory pilot study evaluating a composite intervention, the study design evaluated the collective impact of the video package (dance coupled with clinical messaging) rather than isolating the independent statistical contribution of only the dance component. Videos were produced with the assistance of a professional videographer. Professional dancers performed choreographed routines to music, synchronised with visual displays of symptoms and information about myopia, cataracts, glaucoma, and general eye health to promote awareness and dispel common myths. The educational content incorporated into the videos was developed by the research team, comprising optometrists and eye health researchers, based on current evidence and established eye health guidelines. Key messages on the symptoms, risk factors, prevention, and management of myopia, cataracts, glaucoma, and general eye health were adapted from internationally recognised eye health resources and the existing scientific literature. The content was reviewed by experienced optometrists to ensure scientific accuracy, clinical relevance, and suitability for the Nigerian context before video production. Video production utilised digital cameras, tripods, and LED lighting to ensure high-quality visual and audio output. The videos were randomly assigned to participants using a random number generator, minimising selection bias. Allocation concealment was maintained through third-party randomization. Balance checks across demographic groups confirmed equal distribution of video exposure..

### Data collection instruments

Data were collected using structured pre- and post-intervention questionnaires hosted on Google Forms and administered electronically via tablets and iPads by a trained research assistant. The questionnaires were developed based on validated instruments from prior studies [32–36] and adapted for cultural relevance and comprehension within the Nigerian context.

The questionnaire included four sections including: demographics (Age, gender, education level, occupation, and residence), knowledge assessment (multiple-choice and Likert-scale items evaluating awareness of target eye conditions and myths), attitudes and Perceptions using a rating scale and open-ended questions exploring beliefs about eye health and practices and health-seeking behaviours consisting of items on adherence to recommendations, barriers to care, and intentions for future eye health practices. A pilot test with 20 participants assessed clarity, cultural appropriateness, and technical feasibility. Minor modifications were made based on feedback. Internal consistency of the questionnaire was assessed using Cronbach’s alpha [40], and reliability checks were performed during the pilot. The final questionnaire (shown in Supplementary File S2 Table) demonstrated that Cronbach’s alpha coefficient was satisfactory, α = 0.761.

Regarding the self-reported diagnostic categories shown in Fig 1 (e.g., myopia, presbyopia, astigmatism, conjunctivitis, hyperopia, cataract, age-related macular degeneration, chalazion, and ocular superinfection), these reflect participants’ own selections from a predefined list of terms embedded in the questionnaire; the terms were presented in English, and the research assistant administering the survey was available to clarify any terms participants did not understand upon request. However, comprehension of each individual diagnostic term was not formally assessed prior to selection. It is therefore possible that some participants, particularly those with primary or no formal education, may have had difficulty accurately identifying or recalling a specific clinical diagnosis, raising the possibility of under-reporting or misclassification of diagnoses in this subgroup. This is acknowledged as a limitation of the self-reported diagnostic data.

**Fig 1:**
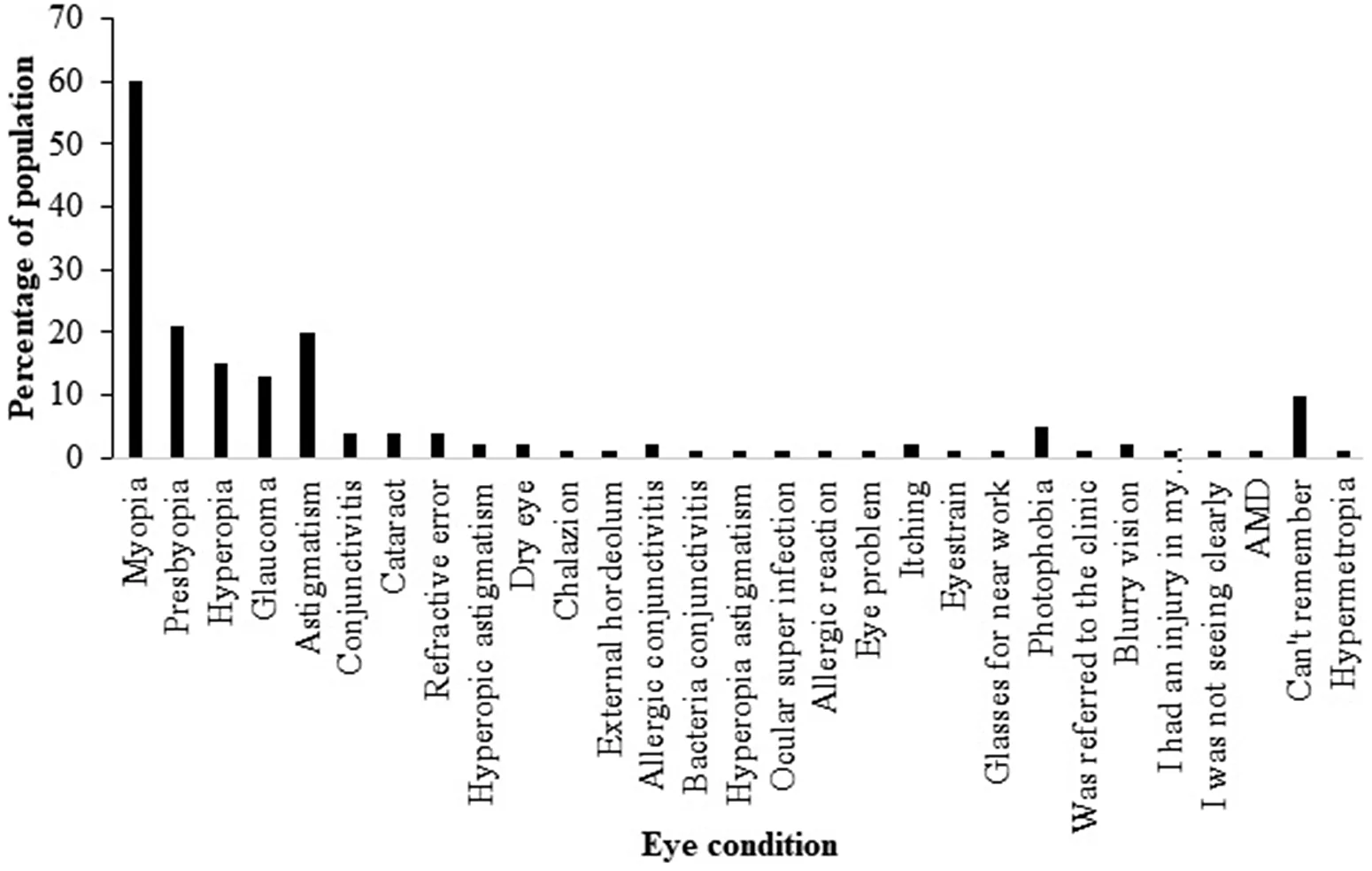
Percentage distribution (%) of self-reported diagnosis among the survey respondents.

### Study procedure

Upon arrival at the clinic, eligible participants were informed about the study and provided written informed consent. To minimise selection bias, participants were randomly exposed to at least one of the four culturally tailored, dance-integrated educational videos covering common eye health topics: myopia, cataract, glaucoma, and myths. While the study design allowed participants to view more than one video if they wished, most participants viewed a single video during the study period. Participants watched these videos in a quiet room. Multiple exposures were recorded when participants voluntarily watched additional videos, and these instances were analysed separately to assess the effect of repeated exposure on knowledge, attitudes, and behavioural intent. This approach preserved random assignment while also capturing the reinforcing effect of repeated viewing, consistent with principles of health education that highlight the importance of repeated message exposure for behaviour change [41–43]. Under a researcher's supervision, all participants completed a post-intervention survey immediately after viewing the videos, measuring knowledge, perceived knowledge, protective behaviours, and intention to attend eye check-ups. Subgroup analyses were conducted based on educational attainment and the number of videos watched.

### Data management and analysis

Survey data were exported from Google Forms into Microsoft Excel 2016 for cleaning and then imported into SPSS version 25.0 [44] for analysis. Descriptive statistics summarised demographic characteristics and baseline responses. In paired categorical data (pre-post comparisons), McNemar’s tests were utilised, whereas paired t-tests were utilised on continuous variables [44]. Since several subgroup analyses have been done (age, gender, education, occupation, geopolitical zone) a Bonferroni correction was done to check Type 1 inflation of error. The level of statistical significance was established at p < 0.05. Missing data were addressed using listwise deletion, and outliers were checked before analysis. For cross-tabulations with sparse cell counts (<5 expected counts in >20% of cells; Tables 3 and 5), Monte Carlo estimates of the exact Fisher–Freeman–Halton p-value (based on 200,000 sampled tables) were calculated for each demographic comparison*.* Data were stored securely and anonymised for confidentiality.

### Ethical considerations

Ethical approval was granted on the 20^th^ January 2025 by the Research and Ethics Committee of the Faculty of Optometry, University of Benin, Nigeria (Reference: EC/UBEN/LSC.OPT/25/50). Participation was voluntary, with all participants providing written informed consent before data collection. Confidentiality was maintained by anonymising data and securing electronic records with password protection. Participants were told that they could withdraw from the study at any time without penalty.

## Results

### Participant demographics and baseline characteristics

A summary of the baseline characteristics of the participants is presented in Table 1. *N* = 438 participants completed the questionnaire, comprising mostly young adults (77.7%, aged 18–44 years), and with higher educational attainment (75.1% possessing tertiary education). The 18–44 year band was collapsed into a single “young adults” category for descriptive summary purposes, consistent with groupings commonly used in health literature; the more granular standardized age categories (18–24, 25–34, 35–44, 45–54, 55 years and above) are disaggregated in Table 1 and were retained for all subgroup and chi-square analyses. The majority of participants were either employed (42.0%) or self-employed (30.4%), with 23.3% identifying as students. Respondents varied significantly by their geographical zone, with the South-South zone contributing the largest proportion of the respondents (54.3%), followed by the North-Central zone (23.1%).

**Table 1:** Participants’ demographic characteristics (n = 438).

Variable	Group	Proportion (%)
Age	18-24 years	127 (29.0)
	25-34 years	119 (27.2)
	35-44 years	94 (21.5)
	45-54 years	70 (16.0)
	55 years and above	28 (6.4)
Gender	Female	230 (52.5)
	Male	208 (47.5)
Educational level	Higher education	329 (75.1)
	Secondary school	80 (18.3)
	Primary/No formal education^α^	29 (6.6)
Occupation	Employed	184 (42.0)
	Self-employed	133 (30.4)
	Unemployed^β^	121 (27.6)
Geopolitical zone	North-West	7 (1.6)
	North-East	4 (0.9)
	North-Central	101 (23.1)
	South-West	34 (7.8)
	South-East	36 (8.2)
	South-South	238 (54.3)
	N/S	18 (4.1)

N/S – Not stated; ^α^Primary Education – 18; ^α^No formal education – 11; ^β^Student – 102; ^β^Unemployed – 15; ^β^Retired - 4

### Baseline knowledge and awareness

Out of the total participants at baseline, *N =* 270 (61.6%) reported having knowledge about eye health and having ever visited an optometry clinic, while 168 (38.4%) had never attended an eye clinic. Regarding diagnosis, *N* = 181 (41.3%) reported being diagnosed with at least one eye condition, whereas 257 (58.7%) reported no diagnosis (p = 0.001).

Among participants who reported being diagnosed with an eye condition, the most common diagnoses were myopia, presbyopia, astigmatism, and hyperopia (Fig 1). Less frequently reported conditions included cataract, age-related macular degeneration (AMD), chalazion, and hypermetropia.

### Knowledge of eye health conditions

Before viewing the educational video, participants were asked to rate their knowledge of eye health issues. The results are shown in Fig 2, indicating that most participants perceive themselves as having a fair to good understanding of eye health, while relatively few consider themselves to be highly knowledgeable (N = 53, 12.1%) or to have very poor awareness (N = 53, 12.1%).

**Fig 2:**
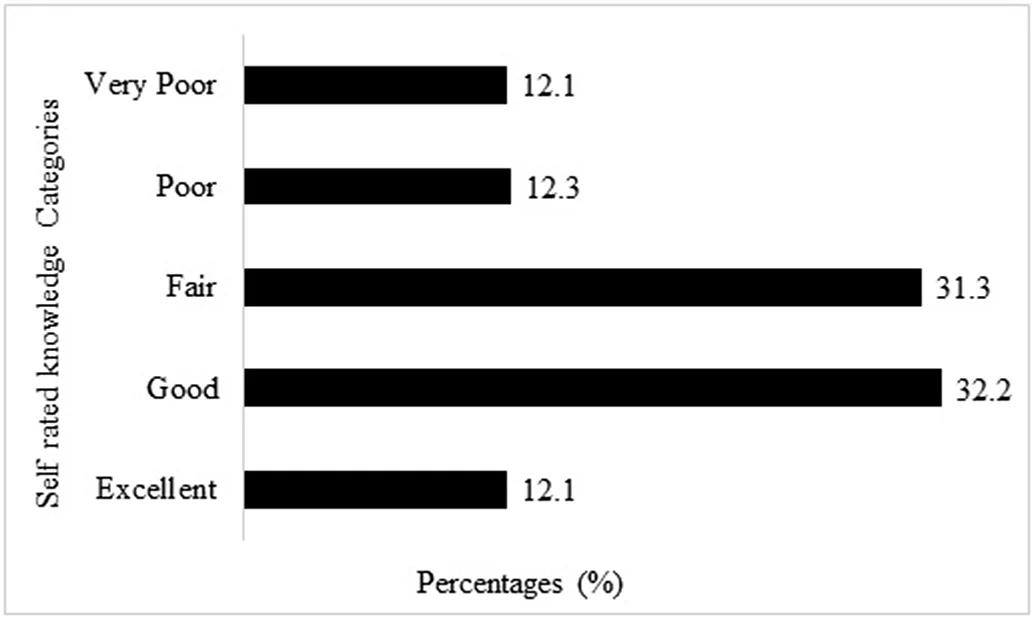
Self-rated baseline knowledge of eye conditions (*N* = 438; Percentages sum to ≤100%).

In terms of awareness of specific eye conditions, the most commonly recognised conditions were cataract (*N =* 254, 58.0%), glaucoma (*N =* 247, 56.4%), and myopia (*N =* 228, 52.1%). Fewer participants were aware of diabetic retinopathy (*N =* 105, 24.0%).

### Beliefs about eye check frequency and the importance of regular eye examinations

Participants were asked how often adults should have their eyes checked by a professional. The most common response was “once a year” (*N =* 153, 34.9%), followed by “very often” (*N =* 121, 27.6%). A smaller proportion reported “I don’t know” (*N =* 73, 16.7%), “only when experiencing issues” (*N =* 60, 13.7%), or “every two years” (*N =* 31, 7.1%).

Chi-square analysis revealed a statistically significant association between beliefs about eye check frequency and education level (χ² = 74.21, p < 0.001). Further, Chi-square analysis demonstrated a significant association between eye check frequency and age (χ² = 26.42, p = 0.048), but no significant association between the importance of regular eye examinations and age (χ² = 10.85, p = 0.21).

### Beliefs about prevention and management of eye conditions, and the importance of regular eye examinations

When asked whether eye conditions can be prevented or managed with proper care, the vast majority of respondents (*N =* 359, 82.0%) answered “yes.” A smaller proportion were uncertain (*N =* 73, 16.7%), and only *N =* 6 participants (1.4%) believed that eye conditions cannot be prevented or managed Chi-square analysis revealed a highly significant association between education level and belief in prevention/management (χ² = 54.86, df = 4, p < 0.001), such that having a higher educational qualification was associated with better prevention or management of eye conditions. Participants’ age was not associated with this outcome (p = 0.875).

Regarding the perceived importance of regular eye check-ups, most participants rated them as “very important” (*N =* 349, 79.7%), with *N =* 80 (18.3%) considering them “somewhat important,” and only *N =* 9 (2.1%) rated them as “not important.” These results underscore widespread recognition of the value of routine eye examinations in promoting and protecting eye health.

### Belief in having enough knowledge and the likelihood of acting

When asked whether they believed they had enough knowledge about eye health, less than one-third of participants (138, 31.5%) responded “yes,” while a larger proportion (201, 45.9%) answered “no,” and 99 (22.6%) were “not sure.” Chi-square analysis indicated a significant association between perceived knowledge and age (χ² = 15.623, p < 0.05) and perceived knowledge and educational level (χ² = 40.315, p < 0.001). However, no significant association was observed with gender (χ² = 0.373, p = 0.830) (Table 2).

**Table 2:** Comparison between demographic data and knowledge of eye health.

Demographic variable	Group	Not sure	No	Yes	χ^2^	p-value
**Age**	18-24 years	22.0%	50.4%	27.6%	15.623	0.048*
	25-34 years	16.0%	42.9%	41.2%		
	35-44 years	24.5%	42.6%	33.0%		
	45-54 years	31.4%	44.3%	24.3%		
	55 years and above	25.0%	53.6%	21.4%		
**Gender**	Female	23.0%	43.9%	33.0%	0.373	0.830
	Male	22.1%	48.1%	29.8%		
**Education**	Primary/No formal education	24.1%	69.0%	6.9%	40.315	<0.001*
	Secondary school	23.8%	65.0%	11.3%		
	Higher education/university	22.2%	39.2%	38.6%		

When asked about willingness to take action, most participants reported that they were “very likely” (341, 77.9%) to seek professional help if they experienced vision problems. A smaller proportion were “somewhat likely” (74, 16.9%), and only 23 (5.3%) were “not likely.” The distribution was highly skewed toward the highest level of action likelihood, demonstrating strong intent among participants to act promptly on vision concerns.

Education was significantly associated with willingness to take action (χ² = 61.64, p < 0.001). By contrast, gender showed no significant influence on likelihood to take action (χ² = 1.43, p = 0.490). While most respondents demonstrate a strong willingness to act if faced with vision problems, their confidence in having sufficient knowledge about eye health remains low. Education level had the strongest association with perceived knowledge and proactive health-seeking behaviour (Table 3).

**Table 3:** Comparison between demographic data and likelihood of acting.

Demographic variable	Group	Not likely	Somewhat likely	Very likely	χ^2^	p-value
**Age**	18-24 years	2.4%	16.5%	81.1%	4.957	0.762
	25-34 years	6.7%	16.0%	77.3%		
	35-44 years	7.4%	16.0%	76.6%		
	45-54 years	5.7%	18.6%	75.7%		
	55 years and above	3.6%	21.4%	75.0%		
**Gender**	Female	5.7%	13.9%	80.4%	1.429	0.490
	Male	4.8%	20.2%	75.0%		
**Education**	Primary/No formal education	3.4%	58.6%	37.9%	61.643	<0.001*
	Secondary school	11.3%	30.0%	58.8%		
	Higher education/university	4.0%	10.0%	86.0%		

*Statistically significant (p < 0.05). Monte Carlo exact Fisher–Freeman–Halton test confirmed the findings.

Exact p-values confirmed the asymptotic Chi-square results: likelihood of acting was not associated with age (exact p = 0.779) or gender (exact p = 0.209) but remained strongly associated with education (exact p < 0.001) Table 3.

### Frequency of eye clinic visits

When asked how often they visited an eye clinic for a check-up, the majority of participants reported attending clinics only when they experienced symptoms (174, 39.7%, Fig 3). Nearly a third stated they never visited an eye clinic (125, 28.5%), while 93 participants (21.2%) reported attending once a year, and only 46 (10.5%) described their visits as very frequent.

**Fig 3:**
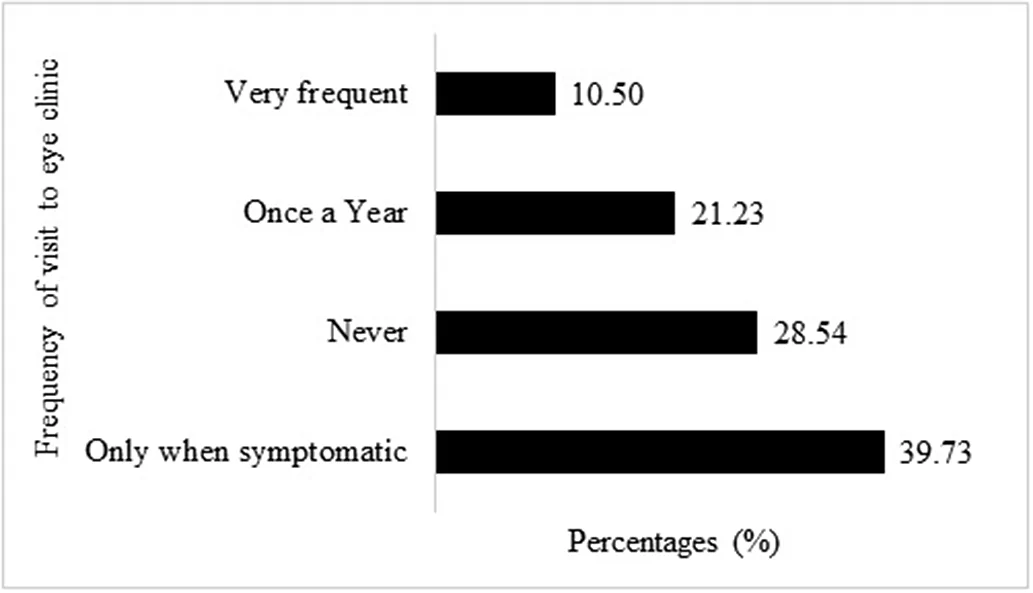
Frequency of eye clinic visits among the respondents (*N* = 438; Percentages sum to ≤100%).

Cross-tabulation by gender revealed important differences in clinic attendance patterns. Males were more likely to report never visiting an eye clinic (79 vs. 46 females), whereas females were more likely to attend once a year (54 vs. 39 males) or only when symptomatic (101 vs. 73 males). Very frequent visits were also slightly higher among females (29 vs. 17 males). Chi-square testing confirmed that age, educational level and gender were significantly associated with clinic visit frequency (p < 0.05) (Table 4).

**Table 4:** Comparison between demographic data and clinic visitation for check up.

Demographic variable	Group	Never	Once a year	Only when I have symptoms	Very frequent	χ^2^	p-value
**Age**	18-24 years	29.9%	15.0%	41.7%	13.4%	21.368	0.045*
	25-34 years	31.1%	20.2%	37.8%	10.9%		
	35-44 years	24.5%	30.9%	39.4%	5.3%		
	45-54 years	31.4%	22.9%	37.1%	8.6%		
	55 years and above	17.9%	17.9%	46.4%	17.9%		
**Gender**	Female	20.0%	23.5%	43.9%	12.6%	8.019	0.046*
	Male	38.0%	18.8%	35.1%	8.2%		
**Education**	Primary/No formal education	51.7%	13.8%	34.5%	0.0%	41.442	<0.001*
	Secondary school	56.3%	12.5%	22.5%	8.8%		
	Higher education/university	19.8%	24.0%	44.4%	11.9%		

* significant difference

### Perception of eye protective measures

When asked whether they take measures to protect their eyes, such as wearing sunglasses or limiting screen time, the majority of respondents (n = 273, 60.9%) reported that they engage in protective behaviours. A substantial number of participants (n = 162, 35.9%) indicated that they do not take any measures, while very few answered “Rarely,” or “Sometimes” (n = 1 each, 0.2%). Protective behaviours were not associated with age (exact p = 0.515) but were significantly associated with gender (exact p < 0.001) and education (exact p < 0.001) Table 5.

**Table 5:** Comparison between demographic variables and perception of eye protective measures.

Demographic variable	Responses						
Age	No	Rarely	Both stated	Sometimes	Yes	χ^2^	p-value
18-24 years	37.0%	0.8%	0.0%	0.0%	62.2%	15.137	0.515
25-34 years	33.6%	0.0%	0.0%	0.0%	66.4%		
35-44 years	38.3%	0.0%	1.1%	0.0%	60.6%		
45-54 years	38.6%	0.0%	0.0%	1.4%	60.0%		
55 years and above	42.9%	0.0%	0.0%	0.0%	57.1%		
**Gender**							
Female	26.5%	0.4%	0.4%	0.4%	72.2%	11.204	0.024*
Male	48.6%	0.0%	0.0%	0.0%	51.4%		
**Education**							
Primary/No formal education	72.4%	0.0%	0.0%	0.0%	27.6%	63.954	<0.001*
Secondary school	73.8%	0.0%	0.0%	0.0%	26.2%		
Higher education/university	24.9%	0.3%	0.3%	0.3%	74.2%		

* Statistically significant (p < 0.05). Monte Carlo exact Fisher–Freeman–Halton test confirmed the findings

### Impact of dance-integrated video on knowledge

Fig 4 presents the number of dance videos watched by the respondents. The most frequently viewed video was “Common Myth about the Eye,” with 27.4% of respondents reporting having watched it. This was followed by the “Myopia” video (26.7%), “Glaucoma” (21.2%), and “Cataracts” (18.0%). Fewer participants reported watching combinations of videos.

**Fig 4:**
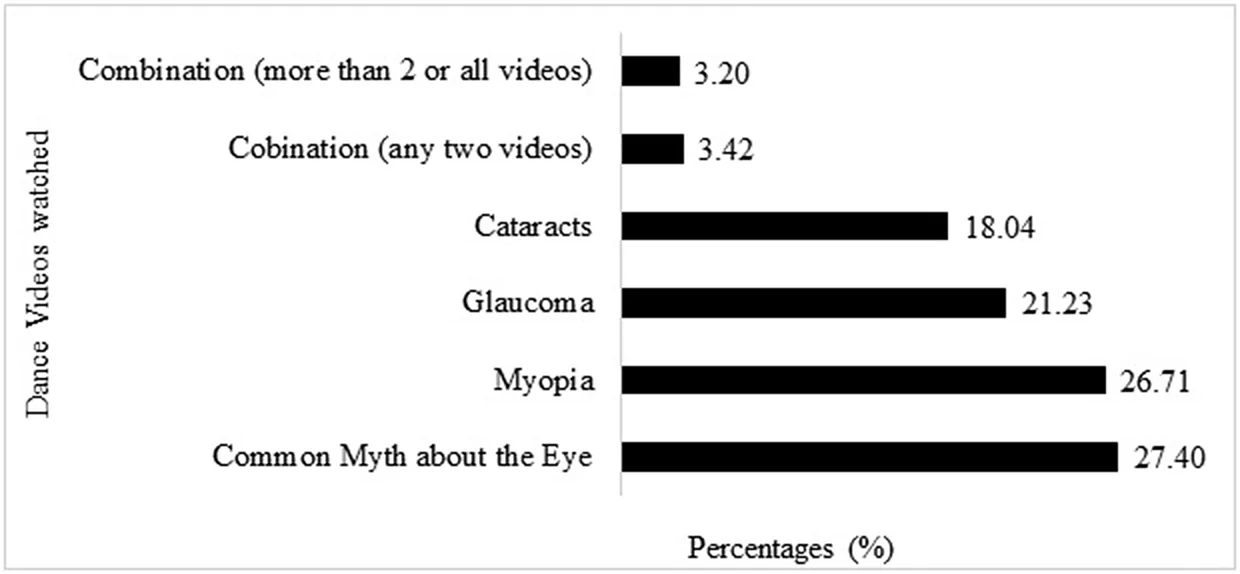
Percentage distribution of the respondents by the dance videos watched (*N* = 438; Percentages sum to ≤100%).

### Evaluating the impact of videos on measured variables

Fig 5 presents the percentage distribution of the participants’ responses pre- and post-video viewing. Pre-video knowledge, which was fair, became good post-video. The intervention was associated with an increase in self-rated knowledge, with the proportion feeling adequately informed increasing by 26.0 percentage points (representing a 61.9% relative increase*,* from 42.0% to 68.0%; *McNemar’s* $\chi^{\text{2}}=\text{6}.\text{145},\text{p}=\text{0}.\text{013}$*, Cramér’s* V = 0.279*).* Self-reported intentions to adopt protective practices (e.g., wearing sunglasses, reducing screen time, and scheduling check-ups) following the intervention (*p* < 0.001) indicated a strong effect size (Cramer’s *V* = 0.118).

**Fig 5:**
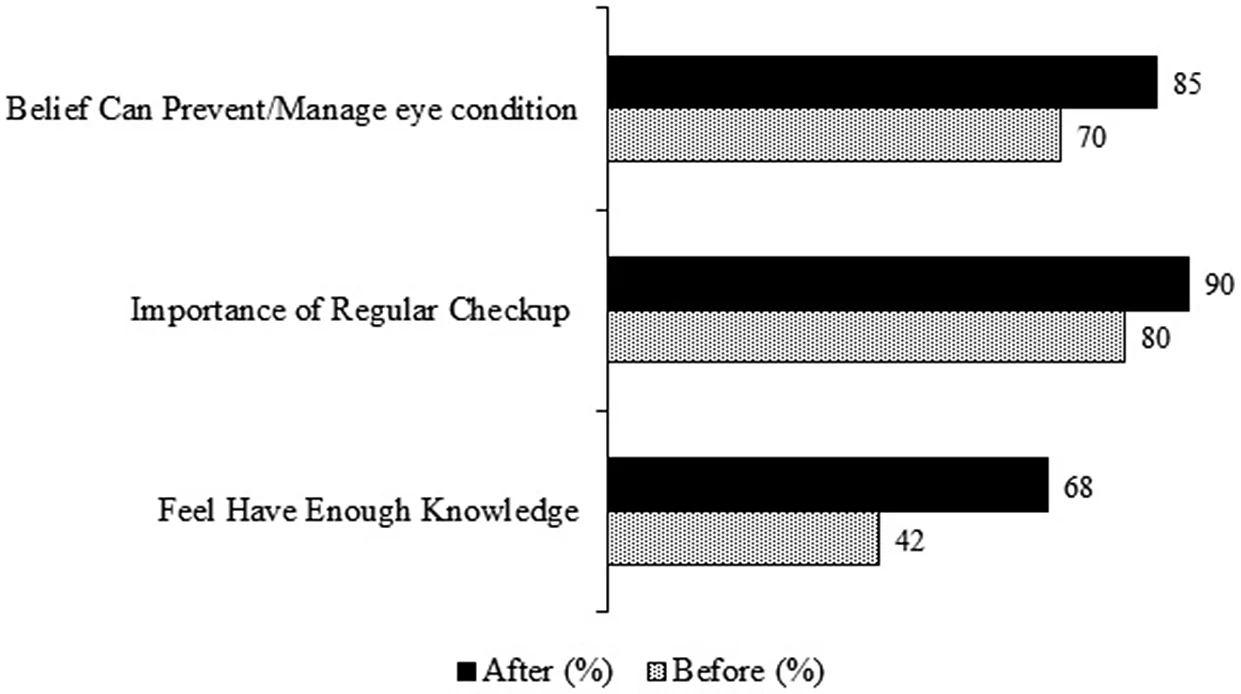
Distribution of participants’ responses before and after exposure to the dance video (*N* = 438; Percentages sum to ≤100%).

The perceived importance of regular checkups and the belief that eye conditions can be managed were also increased, though not statistically significantly within subgroups for the latter (Fig 5). For the question “Can eye conditions be prevented/managed?”, baseline attitudes were already high (70.0% pre-intervention, 85.0% post-intervention), consistent with a ceiling effect in this particular outcome. Consequently, overall post-intervention improvements were statistically significant (p = 0.01; Fig 5).

Table 6 presents the change in attitude before and after participants watched the educational videos with respect to having regular checkups. Exposure to the educational videos was associated with a positive change in attitudes toward regular eye check-ups across most demographic groups. Participants aged 18–54 years indicated significant effects in willingness (93–98 post-intervention, *p* < 0.001). It was also significant in both males and females (95–97%, *p <* 0.001).

**Table 6:** Percentage change in attitude to regular eye checkup post-video, based on demographic variables.

Variable	Number	Yes, to regular eye check-up (Pre)	Yes, to regular eye check-up (Post)	McNemar value	p-value
**Age**					
18-24 years	127	61.4%	97.6%	0.972	0.003
25-34 years	119	62.2%	93.3%	0.891	0.013
35-44 years	94	53.2%	95.7%	0.994	<0.001
45-54 years	70	62.9%	98.6%	1.023	0.005
55 and above	28	85.7%	100%	0.962	0.305
**Gender**					
Female	230	67.0%	97.4%	0.940	0.019
Male	208	55.8%	95.2%	0.988	0.002
**Education Level**					
Higher education (college/univ)	329	71.7%	96.4%	0.945	0.064
Primary/No formal education	29	41.4%	96.6%	1.091	<0.001
Secondary school	80	27.5%	96.3%	0.948	<0.001
**Occupation**					
Employed	184	61.4%	97.3%	0.998	0.004
Self-employed	133	62.4%	97.0%	0.984	0.006
Unemployed	121	61.2%	94.2%	0.884	0.008
**Geopolitical Zones**					
North Central	101	46.5%	100%	–	<0.001
North-East	4	75.0%	100%	–	0.059
North-West	7	85.7%	100%	–	0.305
South-East	36	47.2%	94.4%	0.895	<0.001
South-South	238	71.0%	94.5%	0.932	0.062
South-West	34	47.1%	97.1%	0.944	<0.001
N/R	18	66.7%	100%	0.962	0.011

N/R -Not Reported

The intervention demonstrated its most profound impact among participants with lower educational attainment. While participants with tertiary education showed high baseline willingness for check-ups (71.7%), those with secondary education jumped from 27.5% to 96.3% (*p* < 0.001), and those with primary or no formal education rose from 41.4% to 96.6% (*p* < 0.001). This represents a near-complete erasure of the baseline ‘knowledge-intention gap’ between different education levels following the intervention. There was also a significant improvement between occupational groups and the North-central, South-east, and South-west (*p* ≤ 0.008). Participants reported significantly greater intentions to adopt protective eye health practices immediately following exposure to the educational videos (*p* < 0.001).

### Association between the number of videos watched and survey responses

Watching more videos was associated with even stronger positive attitudes and behavioural intent. Viewers who watch >1 video consistently reported higher percentages for understanding, confidence, and plan to share video information. Notably, all who watched >1 video plan to adopt protective eye practices and share information with others, and the main knowledge rating improved, and their understanding of the various eye conditions increased from 93.2 – 96.4% (Fig 6)

**Fig 6:**
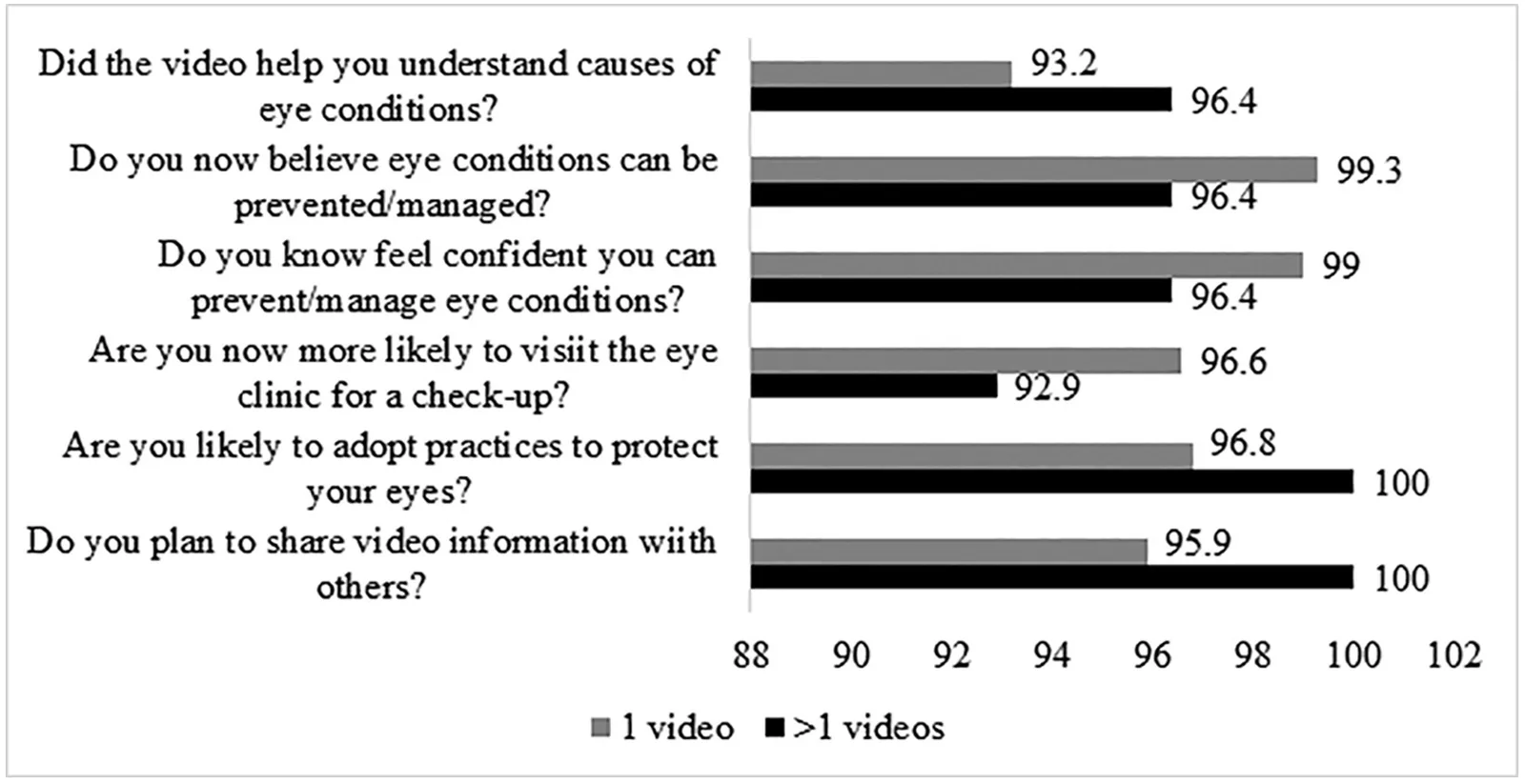
Association between the number of videos watched and participants’ responses to related items (*N* = 438; Percentages sum to ≤100%).

## Discussion

The main aim of this study was to explore the potential of culturally tailored, dance-integrated educational videos in improving knowledge, perception, and preventive behaviours related to eye health among adults in Nigeria. In general, the intervention was associated with improvements in several areas, such as noticeable increases in understanding of eye health and self-perceived knowledge, along with the self-reported adoption of protective behaviours. Importantly, the dance-based intervention achieved these gains despite a highly educated and health-aware baseline population. However, the intervention produced the greatest impact among participants with lower educational attainment, where willingness to attend regular eye check-ups rose dramatically from below 40% to over 95%. These results highlight the intervention’s scalability and potential as an equitable health promotion strategy capable of bridging health literacy gaps and strengthening preventive eye care behaviours in diverse and resource-limited populations. This study should be considered a pilot or exploratory intervention, demonstrating the feasibility and acceptability of the approach and providing preliminary evidence of short-term knowledge gain.

Dance, as a universally accessible form of art, has been leveraged in health campaigns to attract attention, enhance recall, and foster community-level diffusion [32–36,45,46]. In this study, the dance-integrated educational intervention was associated with improvements in eye health knowledge and participants’ self-reported behavioural intentions immediately following exposure, even among an already educated and health-conscious population. Although baseline knowledge of common refractive errors and age-related ocular conditions was relatively high, awareness of diabetes-related eye diseases remained limited. This suggests that there are domain-specific knowledge gaps in health, even among health-literate groups. We acknowledge that the high baseline engagement of our sample, 75.1% of whom possessed tertiary qualifications, along with our recruitment strategy primarily through optometry clinics, captured a predominantly ‘health-seeking’ cohort. This baseline engagement explains the ceiling effect observed in the intention to visit a clinic, as noted previously [47], which was already high at 80% before the intervention began. Because the majority of participants were already proactive about their eye health, the potential for the intervention to produce a large, statistically significant increase in this specific metric was mathematically restricted. This statistical phenomenon likely influenced our effect size estimates for health-seeking intention, resulting in the modest 10% increase observed, in contrast to the more substantial gains achieved in areas where baseline knowledge was lower, such as among participants with primary or no formal education.

An important finding of this study was the relationship between overall behavioural intent and subgroup responsiveness. While the intervention successfully led to statistically significant improvements in general eye health knowledge and willingness to adopt specific protective behaviours, the overall measure for intention to visit an eye clinic increased only modestly (from 80.0% to 90.0%), a change that was not statistically significant (McNemar’s test, p = 0.443).

Conversely, knowledge scores were significantly improved and perceived knowledge increased significantly by 42% to 68%. Besides, self-reported readiness to adopt protective eye health practices, including wearing of sunglasses, screen time and frequent checkups, also exhibited a significant change in the post-intervention period. Such results indicate that culturally based and performance-based media can deliver action-oriented ocular health information even to health-literate and care-seeking people [45].

It is essential to distinguish the observed statistical significance from the practical public health relevance of the intervention. While the overall effect sizes for knowledge gain were modest (Cramer’s V ≈ 0.07), such results must be interpreted within the context of the study's health-seeking and highly educated baseline population. The ‘modest’ nature of the overall effect is a mathematical byproduct of the ceiling effect, where participants already possessed high baseline awareness. From a public health perspective, the practical relevance is most clearly demonstrated in the subgroup analyses, where the intervention bridged massive literacy gaps for the most vulnerable learners. For scalable, art-based health communication, even ‘small’ shifts in broader population knowledge can lead to significant cumulative reductions in avoidable blindness when implemented at scale.

The study also found a statistically significant association between beliefs about eye examinations and educational attainment, indicating that higher education levels positively influenced perceptions about the need for regular eye checks. This finding is consistent with previous research that has reported a significant association between education level and the likelihood of seeking regular eye exams [48,49]. Similarly, educational attainment was strongly associated with better prevention and management of eye conditions, highlighting the central role of education in fostering positive health perceptions and proactive health behaviours [50].

Despite the overall high baseline awareness, the present study revealed a persistent gap in public confidence and awareness regarding personal eye health. A majority of respondents reported limited information about their eye status, irrespective of gender and age, emphasising the need for improved eye health education, consistent with previous research [51]. Notably, participants indicated a willingness to seek care primarily when symptoms arose, with women showing a greater inclination toward clinical visits than men. This contrasts with previous studies that have shown that women’s utilisation of eye care services was lower than that of men [52–54].

Regarding protective eye health measures, most participants were already engaged in preventive practices such as wearing sunglasses and reducing screen exposure. In this study, we found significant associations between these behaviours, gender, and education level. However, participants reported stronger intentions and greater willingness to engage in preventive eye health practices immediately after viewing the educational videos. These results underscore the strength of the intervention in transcending literacy barriers and effectively conveying complex health messages through culturally resonant visual media. The pronounced effects among this group highlight the positive impact of health literacy in empowering people to take action regarding their health at both the individual and community level [18].

Regarding the dynamics of message transmission, our data indicated that exposure to multiple videos was associated with stronger gains in confidence and understanding, and an increased likelihood of information-sharing among participants. This supports the “repetition principle” in health education, which emphasises that repeated exposure enhances message retention and behavioural change [55]. It is also important that repetitive activities targeted at behavioural change should be fun and engaging [56,57]. Therefore, dance, as a vehicle for health education, is particularly powerful in communal, oral, and participatory cultures where it functions as both entertainment and social commentary [58,59]. It is important to highlight that the integration of culturally relevant motifs and familiar rhythms in our study likely amplified message resonance and engagement, consistent with literature emphasising the effectiveness of culturally tailored health communication in improving message adoption and health outcomes [60,61]. The especially strong responsiveness among less-educated groups indicates that culturally resonant, performance-based strategies do not merely distribute information; they actively contribute to equity in health-education outcomes. By bridging massive literacy gaps and raising the health-seeking intent of vulnerable learners to the level of their more educated peers, this approach offers a scalable model for closing health knowledge gaps in resource-limited settings.

Beyond its educational utility, the dance-integrated model functions as a vital form of cultural social capital. By utilising familiar rhythms and culturally resonant motifs, the intervention taps into existing communal bonds and collective identities, thereby fostering a sense of trust and social engagement that traditional vertical health messages often lack. In participatory and oral cultures like Nigeria, where performance art serves as a primary vehicle for social commentary, this cultural capital acts as a bridge between clinical health information and community acceptance. To ensure long-term sustainability and scalability, this approach must move beyond stand-alone interventions and be integrated into the broader Nigerian health system. One viable pathway is the formal integration of these art-based materials into the Primary Healthcare framework [62]. Specifically, the videos could be utilised for waiting-room screenings in primary health centres to engage patients during wait times or included as part of digital toolkits for community health workers conducting rural outreach. Such systemic integration would capitalise on the intervention’s proven success in reaching less-educated subgroups, providing a low-cost, high-impact strategy for promoting equitable eye health literacy at a national scale

### Limitations and strengths

There are a few limitations that should be considered when interpreting these findings. First, there is the quasi-experimental design that lacks a control group, which constrains causal inference. Although we have identified potential threats to internal validity (testing effects, social desirability bias, Hawthorne effects), they could not be fully controlled without a control group. This should be included in future studies to prove the causal relationship more firmly. Second, although participant recruitment was initiated during community outreach activities, utilizing optometry clinics as central data collection hubs may have introduced selection bias toward an uncharacteristically health-literate and care-engaged sample, thereby constraining generalizability to individuals with lower baseline health access. Third, outcome measures such as knowledge, behaviours and attitudes are all self-reported, which also presents the possibility of response bias. The effectiveness of the interventions would be better demonstrated by objective measures of real clinic visits and confirmed behavioural changes. Fourth, the multiple subgroup analyses performed on the demographic variables (age, gender, education, occupation, region) contribute to the risk of Type 1 error (false positives). Even though the Bonferroni correction was used to check for Type 1 error inflation, there was a high number of comparisons that could increase the risk of Type 1 error. Fifth, self-rated knowledge had improved, but it needs longer-term follow-up to help in establishing whether the increased willingness leads to actual attendance in the ocular clinic and long-term attention to preventive behaviours. Lastly, the ceiling effect in intention to visit an eye clinic, which was identified, is recognised, though it needs a formal statistical confirmation of the ceiling-effect diagnostics in future research studies. Finally, we must acknowledge a key design limitation: because this study lacked an active, standard-education control group, we cannot statistically isolate the independent effect size of the dance choreography from the factual clinical content. The observed improvements represent the combined impact of this multimodal educational tool. Future RCT designs should compare standard text-based videos against dance-integrated videos to determine the isolated efficacy of the performative arts component. Nonetheless, despite these limitations, there are strengths in this study. One, it has a groundbreaking and culturally relatable strategy, and it takes the form of a dance-based and educational video, which is highly engaging in the culture of Nigerians. Second, the intervention worked especially well when applied to participants with a lower educational level, and the implementation of several videos was associated with a cumulative increase in positive attitudes and behavioural intent. Lastly, the strong influences in some of the most vulnerable student groups underscore the potential of generalizability of this method in managing preventable blindness of various communities in Nigeria.

## Conclusion

In conclusion, this study offers evidence that a culturally tailored, dance-integrated educational intervention was associated with improvements in knowledge, perceived understanding, and immediate self-reported intentions to engage in preventive eye health practices following exposure to the educational videos. The notable impact observed among less-educated subgroups indicates that this method of health communication may be especially effective for closing health knowledge gaps and promoting equitable access to health information. Future initiatives should aim to extend this approach into more diverse populations by incorporating regional dialects, relatable visual narratives, and accessible digital delivery platforms, as well as assessing actual behavioural changes and clinical outcomes, and integrating with broader health systems strategies to maximise the potential for reducing preventable vision loss. The study measures immediate intention and knowledge gain rather than long-term behavioural habits, which remains a focus for future longitudinal research. This study serves as a valuable pilot, demonstrating the feasibility and acceptability of this innovative approach.

## Supporting information

S1 Table Reporting standards checklist for submission: dance-integrated educational videos study. (DOCX)

S2 Table Questionnaire. (PDF)

S1 File Videos. (DOCX)

S1 Data Anonymized data generated from this study. (XLSX)
