# Supplementary material for: Feasibility and immediate effects of a multimodal, dance-integrated educational video package on eye health knowledge and intentions in Nigeria: A pre-post pilot study
Source: PLOS Glob Public Health. 2026 Oct 14;6(10):e0005585. doi: 10.1371/journal.pgph.0005585 (PMC13667777; doi:10.1371/journal.pgph.0005585)
Supplement: Supplementary file 1 [file pgph.0005585.s002.pdf]

S2 Table: Questionnaire

Adapted from the works by **Mills et al**. (2011) and modified to the unique context of this study.

This questionnaire is designed to evaluate the effectiveness of integrating dance and educational messages to raise awareness of eye health in six geopolitical zones in Nigeria.
Your participation in this study is invaluable.
Your participation in this study is entirely **voluntary**.
You have the right to withdraw from the study at any time without facing any consequences.
The information collected will be kept **confidential** and used for **research** purposes only.

By proceeding with the questionnaire, you acknowledge that you have read and understood the information provided in this **consent form**.
Your voluntary participation is appreciated.

* Indicates required question

1. What is your name? *

PRE- INTERVENTION

Demographics

2. **What is your age?** *

Mark only one oval.

- 18 -24 years
- 25 – 34 years
- 35 –44 years
- 45 – 54years
- 55 and above

3. What is your State? *

Mark only one oval.

- 1. Abia State
- 2. Adamawa State
- 3. Akwa Ibom State
- 4. Anambra State
- 5. Bauchi State
- 6. Bayelsa State
- 7. Benue State
- 8. Borno State
- 9. Cross River State
- 10. Delta State
- 11. Ebonyi State
- 12. Edo State
- 13. Ekiti State
- 14. Enugu State
- 15. Gombe State
- 16. Imo State
- 17. Jigawa State
- 18. Kaduna State
- 19. Kano State

- 20. Katsina State
- 21. Kebbi State
- 22. Kogi State
- 23. Kwara State
- 24. Lagos State
- 25. Nasarawa State
- 26. Niger State
- 27. Ogun State
- 28. Ondo State
- 29. Osun State
- 30. Oyo State
- 31. Plateau State
- 32. Rivers State
- 33. Sokoto State
- 34. Taraba State
- 35. Yobe State
- 36. Zamfara State
- FCT-ABUJA

4. **What is your Gender?** *

Mark only one oval.

- Male
- Female
- Other: ____________

5. **What is your level of education?** *

Mark only one oval.

- No formal education
- Primary school
- Secondary school
- Higher education (college/university)

6. **Occupation** *

Mark only one oval.

- Student
- Employed
- Self-employed
- Unemployed
- Retired

7. **Have you ever visited an optometry clinic before today?** *

Mark only one oval.

- Yes
- No

8. **Have you been diagnosed with any eye condition?** *

Mark only one oval.

- Yes
- No

9. If **YES,** please specify.

____

Knowledge of Eye Health(Baseline)

10. **How would you rate your knowledge of eye health issues?** *

Mark only one oval.

- Very Poor
- Poor
- Fair
- Good
- Excellent

11. **Which of the following eye conditions are you aware of? (Select all that apply):** *

Check all that apply.

- [ ] CATARACT
- [ ] GLAUCOMA
- [ ] DIABETIC RETINOPATHY
- [ ] MYOPIA
- [ ] NONE OF THE ABOVE

12. **How often do you believe adults should have their eyes checked by a professional?** *

Mark only one oval.

- Once a Year
- Every two years
- Only when experiencing issues
- Very Often
- I don’t know

13. **Can eye conditions be prevented or managed?** *

Mark only one oval.

- Yes
- No
- Not Sure

Attitude and Perception

14. **How important do you think it is to get regular eye check-ups?** *

Mark only one oval.

- Very Important
- Somewhat Important
- Not Important

15. **Do you believe you have enough knowledge about eye health?** *

Mark only one oval.

- Yes
- No
- Not Sure

16. **How likely are you to take action if you experience vision problems?** *

Mark only one oval.

- Very Likely
- Somewhat Likely
- Not Likely

PRACTICES

17. **How often do you visit an eye clinic for a check-up?** *

Mark only one oval.

- Once a Year
- Only When I have Symptoms
- Very frequent
- Never

18. Do you take any measures to protect your eyes (e.g., wearing sunglasses, and avoiding excessive screen time)? **Others**, **Please Specifiy** *

Mark only one oval.

- Yes
- No
- Other: ______________

Post-Survey Questions

WATCH AT LEAST ONE OF THESE VIDEOS AND NOTE THE NAME OF THE VIDEO(S) YOU WATCHED.

COMMON MYTH ABOUT THE EYE

MYOPIA VIDEO

GLAUCOMA VIDEO

CATARACTS VIDEO

19. **Which of the videos did you watch?** *

Check all that apply.

- [ ] Common Myth about the Eye
- [ ] Myopia
- [ ] Glaucoma
- [ ] Cataracts

20. **Did the video you just watch help you understand the causes of eye conditions like myopia, glaucoma, or cataracts?** *

Mark only one oval.

- ( ) Yes
- ( ) No

21. **Do you now believe the eye conditions can be prevented or managed with proper care?**

Mark only one oval.

- ( ) Yes
- ( ) No
- ( ) Not Sure

22. **How would you rate your understanding of the eye condition(s) presented in the video?**

Mark only one oval.

- Poor
- Fair
- Good
- Excellent

CHANGE IN ATTITUDE

23. **After watching the video, how important do you think it is to get regular eye check-ups?** *

Mark only one oval.

- Very Important
- Somewhat Important
- Not Important

24. **Do you now feel confident that you can take steps to prevent or manage eye conditions?** *

Mark only one oval.

- Yes
- No
- Not Sure

INTENTION TO ACT

25. **Are you now more likely to visit an eye clinic for a check-up?** *

Mark only one oval.

- Yes
- No

26. **Are you likely to adopt practices to protect your eyes (e.g., wearing sunglasses, managing diabetes)?** *

Mark only one oval.

- Yes
- No

27. **Do you plan to share the information you learned from the video with others?** *

Mark only one oval.

- Yes
- No

FEEDBACK ON THE VIDEO

28. Did you find the video engaging and easy to understand? *

Mark only one oval.

- Yes
- No

29. **What did you like most about the video?** *

____
____
____
____
____

30. **Do you have any suggestions for improvement?**
If yes, type below. *

____
____
____
____
____

This content is neither created nor endorsed by Google.

Google Forms
