# Supplementary material for: Feasibility and immediate effects of a multimodal, dance-integrated educational video package on eye health knowledge and intentions in Nigeria: A pre-post pilot study
Source: PLOS Glob Public Health. 2026 Oct 14;6(10):e0005585. doi: 10.1371/journal.pgph.0005585 (PMC13667777; doi:10.1371/journal.pgph.0005585)
Supplement: Supplementary file 2 [file pgph.0005585.s001.docx]

# **Supporting information**

**S1 Table**: Reporting Standards Checklist for Submission: Dance-Integrated Educational Videos Study

| Item | Description of Required Information | Page(s) | Line(s) |
| --- | --- | --- | --- |
| TITLE and ABSTRACT |  |  |  |
| 1. Title | Clearly identify the study design (Cross-Sectional, Pre–Post Interventional Study) and intervention type (Dance-Integrated Educational Videos). | 1, 2 | 1, 8 |
| 2. Background/Objective | State the problem (eye health challenges in Nigeria) and the study aim (evaluated effectiveness in improving knowledge, attitudes, and preventive behaviours). | 1 | 30–34 |
| 3. Study Design | Specify the design type (pre–post interventional study). | 1 | 35 |
| 4. Sample Size | Report the total number of participants (N=438). | 1 | 42 |
| 5. Key Results | Report the main outcome measures and significance (e.g., knowledge improved by 52.4%, p=0.013; perceived knowledge increased by 61.9%, p=0.013). | 1 | 45–47 |
| 6. Conclusion | Summarize the key findings and implications (significant enhancement of knowledge and behaviours, especially in lower-education subgroups). | 2 | 56–61 |
| INTRODUCTION |  |  |  |
| 7. Rationale | Provide context for the intervention, citing cultural barriers, poor literacy, and high burden of avoidable vision impairment in Nigeria. | 3 | 78–95 |
| 8. Scientific Basis | Explain why dance was chosen (culturally embedded, participatory method, previously successful in other health domains). | 4 | 100–111 |
| METHODS |  |  |  |
| 9. Study Design | State explicitly the study design (quantitative pre- and post-intervention quasi-experimental study) and dates (April 11 to June 22, 2025). | 5 | 116–122 |
| 10. Setting | Describe the recruitment setting (optometry clinics in Nigeria’s six geopolitical zones). | 5 | 123–127 |
| 11. Participants | State inclusion criteria (Nigerian adults ≥ 18, able to read/write English, written informed consent) and exclusion criteria (cognitive/hearing impairments). | 5 | 128–136 |
| 12. Sample Size | Detail the method of determination (Fisher’s formula, 50% prevalence, 95% CI, 5% margin of error, target N=424). | 6 | 137–160 |
| 13. Intervention | Describe the core components (four culturally tailored, dance-integrated educational videos; 3 minutes each) and content (myopia, cataracts, glaucoma, myths). | 7 | 163–173 |
| 14. Allocation/Assignment | Explain how participants were assigned to videos (randomly assigned to minimize selection bias and ensure exposure to all topics). | 8 | 189-195 |
| 15. Data Collection Instruments | Describe the collection method (Google forms administered electronically via tablets/iPads) and the sections of the questionnaire (demographics, knowledge, attitudes, behaviours). | 7, 8 | 200–204 |
| 16. Instrument Testing | Report on pilot testing (20 participants) and reliability assessment (Cronbach's alpha *α*=0.761). | 8 | 184–188 |
| 17. Study Procedure | Detail the process: informed consent, viewing videos in a quiet room, immediate post-intervention survey. | 8 | 189–195 |
| 18. Statistical Analysis | List the analytical methods (Descriptive statistics, Paired t-tests, McNemar’s test, Chi-square analysis) and significance level (p<0.05). | 9 | 205–214 |
| 19. Ethical Approval | Provide the approval source and reference number (Research and Ethics Committee, University of Benin, Reference: EC/UBEN/LSC.OPT/25/50). | 9 | 215–221 |
| RESULTS |  |  |  |
| 20. Participant Flow | State the number of participants who completed the study (N=438). | 10 | 225-226 |
| 21. Baseline Data | Report baseline demographics (Age, Gender, Education, Occupation, Geopolitical Zone). | 10, 11 | 232–234 |
| 22. Outcome Data (Knowledge) | Report specific measures of change in knowledge (61.9% increase in self-rated knowledge, p=0.013). | 1, 20 | 45, 355 |
| 23. Outcome Data (Behavioural Intent) | Report measures of change in specific behaviours (Adoption of protective practices improved markedly, p<0.001). | 1, 20 | 49, 358 |
| 24. Subgroup Analyses | Report the findings of the pre-specified subgroup analysis (e.g., strong impact in lower educational attainment, increasing willingness to attend check-ups from 27.5%/41.4% to over 96%, p<0.001). | 2, 21 | 51–53, 374-375 |
| DISCUSSION |  |  |  |
| 25. Key Results Summary | Summarize the main findings relative to the objective (Dance-based intervention showed clinically relevant effectiveness, despite a high baseline population). | 25 | 399–407 |
| 26. Interpretation/Mechanism | Discuss the implication of the findings (e.g., culturally grounded media can deliver actionable knowledge; repetition enhances message retention). | 26 | 422–426, 456–468 |
| 27. Generalizability | Discuss limitations related to the sample (disproportionate level of education and pre-existing preventive-care engagement limits generalisability). | 28 | 469–476 |
| 28. Limitations & Future Research | Note limitations (modest change in intention to visit clinic, lack of long-term follow-up) and suggestions for future work (extend to diverse populations, assess actual behavioural change). | 28 | 477–488 |
| OTHER INFORMATION |  |  |  |
| 29. Funding | Disclose the funding source (VOSH International provided funding for video production and data collection). | 30 | 509–512 |
| 30. Conflicts of Interest | Declare conflicts (authors declare no conflicts of interest). | 30 | 513–514 |
| 31. Author Contributions | Clearly specify contributions of all listed authors (e.g., supervision, study design, data collection, statistical analysis, manuscript drafting/review). | 30 | 515–526 |
| 32. Supplementary Materials | Reference supplementary files containing links to the videos (SF1 – SF4). |  |  |
