# Supplementary material for: Feasibility and immediate effects of a multimodal, dance-integrated educational video package on eye health knowledge and intentions in Nigeria: A pre-post pilot study
Source: PLOS Glob Public Health. 2026 Oct 14;6(10):e0005585. doi: 10.1371/journal.pgph.0005585 (PMC13667777; doi:10.1371/journal.pgph.0005585)
Supplement: Supplementary file 3 [file pgph.0005585.s003.docx]

**S1 Video: Knowledge about myopia**

<https://drive.google.com/file/d/1mucGhxXvLKOi5vz_X4Nrwzh15IfJIExD/view?usp=drivesdk>

**S2 Video: Knowledge about cataract**

<https://drive.google.com/file/d/1nfUws0BYM-mfjOKo0onasVWRcLSK8H5E/view?usp=drivesdk>

**S3 Video: Knowledge about glaucoma**

<https://drive.google.com/file/d/1Iyv7FfBjzt-sL_6ZuVsqaSWnxV25D8m-/view?usp=drivesdk>

**S4 Video: Dispelling myths about ocular problems**

<https://drive.google.com/file/d/1Ji-hrVTp3_4ekIn2R47XXW5QsTVHzEdq/view?usp=drivesdk>
