# Supplementary material for: Feasibility and immediate effects of a multimodal, dance-integrated educational video package on eye health knowledge and intentions in Nigeria: A pre-post pilot study
Source: PLOS Glob Public Health. 2026 Oct 14;6(10):e0005585. doi: 10.1371/journal.pgph.0005585 (PMC13667777; doi:10.1371/journal.pgph.0005585)
Supplement: Supplementary file 4 [file pgph.0005585.s006.docx]

Response to Reviewers.

Dear Editor,

Thank you for the opportunity to revise our manuscript. We appreciate the reviewers’ insightful comments, which have significantly strengthened the paper. We have addressed all comments to the best of our ability. All revisions made in response to the reviewers’ feedback and sections that were proofread or edited for clarity are highlighted in coloured font in the manuscript. We sincerely appreciate your time and consideration and look forward to the favourable acceptance of our manuscript.

|  | Reviewer’s Comment | Response | Action Take |
| --- | --- | --- | --- |
| Reviewer  1. | (p6) The pre–post quasi-experimental design without a control group limits causal inference. Potential threats such as testing effects, social desirability bias, and Hawthorne effects are not addressed. Authors should discuss these limitations more explicitly or consider adding methodological safeguards. | Thank you for the comments. | This has been addressed in the limitation session, lines 466 to 470. |
| Reviewer 2. | Recruitment exclusively from optometry clinics introduces significant selection bias, resulting in an unusually health-literate and care-engaged sample. This undermines generalizability, especially for populations with low literacy. Authors should discuss sampling alternatives or provide weighting strategies. | This limitation is well acknowledged and has been cited in the manuscript. | This has been addressed in the manuscript session, lines 470 to 474 |
| Reviewer 3 | (pp14-15) The manuscript frequently uses Chi-square tests for pre–post comparisons even when responses are paired. The appropriate test for paired categorical data is McNemar’s test, yet this is inconsistently applied. Authors should revise all analyses to ensure correct test–data alignment | This has been done, and the result presented using McNemar | Changes have been effected in Table 2. See lines 353 to 379. |
| Reviewer 4. | (pp15-17) The manuscript reports percentage improvements (e.g., 52.4% improvement) without defining the formula used. Absolute vs. relative change calculations should be distinguished. Effect sizes (Cohen’s d, Cramer’s V) would provide better interpretability. | The changes have been revised in the manuscript | See lines 44 to 47 and lines 353 to 358. |
|  |  |  |  |
| 5. | pp16-18) Although the authors mention a ceiling effect, it is not statistically verified. Pre-intervention distributions show skewed high-intention values, which should be analysed using ceiling-effect diagnostics or sensitivity tests. | Thank you for this observation. We agree that the high pre-intervention intention scores suggest a potential ceiling effect. While formal ceiling-effect diagnostics were not conducted, we have clarified this in the revised manuscript. The pre-intervention distribution was highly skewed toward strong baseline intention (80% reporting “very likely” to visit an eye clinic), leaving limited statistical room for detectable improvement. As a result, even meaningful behavioural shifts may not have reached statistical significance in the pre–post comparison | See lines 416 to 418 |
| 6. | (pp 18-20) All outcomes—including knowledge, behaviours, and attitudes—are self-reported, raising the risk of response bias. Authors should discuss this limitation explicitly and consider recommending objective behavioural measures (e.g., actual clinic attendance). | Thank you for highlighting this important point. We agree that the exclusive use of self-reported outcomes introduces the potential for response bias, including social desirability and recall bias. In the revised manuscript, we have explicitly acknowledged this as a limitation. Because participants self-reported their knowledge, attitudes, and behavioural intentions, their responses may not fully reflect actual behaviour or long-term practice change | Line 447 to 450; see line 458 to 450 |
| 7. | (pp 20-22) Numerous subgroup analyses are conducted (age, gender, education, region), increasing Type I error risk. The manuscript does not mention correction methods (Bonferroni, Holm). Authors should adjust p-values or limit the number of comparisons. | A Bonferroni correction was used to check for Type 1 error inflation (Page 9, L209). However, in the Limitations section (Page 28, L485–487) we have still maintained the statement that even with the Bonferroni correction, the high number of comparisons contributes to the risk of Type 1 error. | See lines 206 to 212. |
| 8. | (pp23-24) The paper states videos were “randomly assigned,” but provides no description of the randomisation process, allocation concealment, or balance checks. This weakens claims of internal validity. | See revised manuscript. | 168 to 171. |
| 9. | An alpha of 0.989 is unusually high and suggests redundant or overly similar questionnaire items. The authors should examine whether items measure unique constructs or consider item reduction. | See revised manuscript | Line 186 |
| 10. | The discussion occasionally implies causal effects (e.g., “the intervention improved behaviour”) despite the absence of a controlled experimental design. Language should be softened to “associated with” or “may have contributed to | See revised manuscript. | 369 to 373 |
| 11 | (pp3-4) The introduction references the distinction but does not explain how it applies specifically to the intervention. | Thank you for this important observation. We have clarified in the revised manuscript how the distinction between health education and health promotion applies specifically to our intervention. While health education focuses on increasing knowledge and understanding of eye health conditions, health promotion takes a broader approach by using culturally relevant strategies to motivate behaviour change and strengthen community engagement | Answer found in lines 88 to 93 |
| 12. | (p5) The sample size calculation citing “Fisher’s formula” lacks detail (exact equation, assumed prevalence justification). | Well acknowledge | The Fisher formula has been imputed into the manuscript. Found between lines 141 and 158 |
| 13 | p12) Figures (e.g., Fig 4–6) lack information about scales, sample sizes, and statistical notes. | In response to reviewer feedback, Figures 4–6 have been revised to improve clarity and transparency. Specifically, we have added clearly labelled scales on all axes, included sample sizes (n) for each variable or comparison displayed, and provided relevant statistical notes, including p-values and indicators of significance where applicable. These additions ensure that the figures are self-explanatory and conform to recommended reporting standards | It has been affected. in various figs |
| 14. | (p25) Terms such as “eye condition,” “eye disease,” and “ocular condition” are used interchangeably. Standardising terminology would improve clarity. | We prefer eye conditions | This has been replaced. |
| : |  |  |  |
