# Supplementary material for: Feasibility and immediate effects of a multimodal, dance-integrated educational video package on eye health knowledge and intentions in Nigeria: A pre-post pilot study
Source: PLOS Glob Public Health. 2026 Oct 14;6(10):e0005585. doi: 10.1371/journal.pgph.0005585 (PMC13667777; doi:10.1371/journal.pgph.0005585)
Supplement: Supplementary file 5 [file pgph.0005585.s007.docx]

Academic Editor
PLOS Global Public Health

Journal Requirements:

If the reviewer comments include a recommendation to cite specific previously published works, please review and evaluate these publications to determine whether they are relevant and should be cited. There is no requirement to cite these works unless the editor has indicated otherwise. 

Please review your reference list to ensure that it is complete and correct. If you have cited papers that have been retracted, please include the rationale for doing so in the manuscript text, or remove these references and replace them with relevant current references. Any changes to the reference list should be mentioned in the rebuttal letter that accompanies your revised manuscript. If you need to cite a retracted article, indicate the article’s retracted status in the References list and also include a citation and full reference for the retraction notice.

Additional Editor Comments (if provided):

The Editorial Office sincerely thanks the authors for their careful and thorough revisions across two rounds of review.

After consideration of the reviewers’ comments, we invite the authors to revise the manuscript accordingly, taking into full account the points raised by both reviewers. We kindly ask that a detailed, point-by-point response to each comment be provided upon resubmission.

We look forward to receiving your revised manuscript.

**Response**:

Thank you for the opportunity to revise our manuscript. All comments have been addressed in red fonts. In addition, we have conducted extensive proofreading of the article to enhance comprehension of the language.

[Note: HTML markup is below. Please do not edit.]

Reviewers' comments:

Reviewer's Responses to Questions

**Comments to the Author**

1. If the authors have adequately addressed your comments raised in a previous round of review and you feel that this manuscript is now acceptable for publication, you may indicate that here to bypass the “Comments to the Author” section, enter your conflict of interest statement in the “Confidential to Editor” section, and submit your "Accept" recommendation.

Reviewer #1: (No Response)

Reviewer #2: All comments have been addressed

Response: Thank you for the comments

2. Does this manuscript meet PLOS Global Public Health’s [publication criteria](https://url.au.m.mimecastprotect.com/s/jAXNCBNqkvfVM4x6Pf6hKI24V6b?domain=track.editorialmanager.com)? Is the manuscript technically sound, and do the data support the conclusions? The manuscript must describe methodologically and ethically rigorous research with conclusions that are appropriately drawn based on the data presented.

Reviewer #1: (No Response)

Reviewer #2: Yes

Response: Thank you for the comments

3. Has the statistical analysis been performed appropriately and rigorously?

Reviewer #1: (No Response)

Reviewer #2: Yes

Response: Thank you for the comments

4. Have the authors made all data underlying the findings in their manuscript fully available (please refer to the Data Availability Statement at the start of the manuscript PDF file)?

The [PLOS Data policy](https://url.au.m.mimecastprotect.com/s/KsI-CD1vmxcBnV7vjIAi8Ij-O0r?domain=track.editorialmanager.com) requires authors to make all data underlying the findings described in their manuscript fully available without restriction, with rare exceptions. The data should be provided as part of the manuscript or its supporting information, or deposited in a public repository. For example, in addition to summary statistics, the data points behind means, medians and variance measures should be available. If there are restrictions on publicly sharing data—e.g. participant privacy or use of data from a third party—those must be specified.

Reviewer #1: (No Response)

Reviewer #2: Yes

Response: Thank you for the comments

5. Is the manuscript presented in an intelligible fashion and written in standard English?

PLOS Global Public Health does not copyedit accepted manuscripts, so the language in submitted articles must be clear, correct, and unambiguous. Any typographical or grammatical errors should be corrected at revision, so please note any specific errors here.

Reviewer #1: (No Response)

Reviewer #2: Yes

Response: Thank you for the comments

6. Review Comments to the Author

Please use the space provided to explain your answers to the questions above. You may also include additional comments for the author, including concerns about dual publication, research ethics, or publication ethics. (Please upload your review as an attachment if it exceeds 20,000 characters)

Reviewer #1: I would like to commend the authors for their creative and culturally resonant approach to eye health promotion in Nigeria. Utilising dance-integrated videos is an innovative way to bridge the gap in health literacy, particularly in resource-limited settings. The finding that this intervention was especially effective among participants with lower educational attainment is highly encouraging from a public health equity perspective. While the study has clear merits, I believe addressing the following points organized by section would further strengthen the manuscript.

**Response**: Thank you for this very useful comment, which has helped to strengthen our manuscript. We have provided point by point response to each comment and made changes in the manuscript text using red fonts.

1. Introduction

(1) Conceptual Framework: The manuscript distinguishes between health education and health promotion.
- Please elaborate on how the dance component functions specifically as a health promotion mechanism, particularly in motivating behavioural change beyond simple information delivery (e.g., emotional engagement, social modelling, cultural resonance).

**Response:** We thank the reviewer for this suggestion. We have expanded the Introduction to explain that while the videos provide *education* (facts on cataracts/glaucoma), the *dance* component acts as a *health promotion* mechanism by leveraging emotional engagement, cultural resonance, and social modelling to lower psychological barriers to care [See Lines 94 – 97]

(2) Contextual Background: The authors provide a compelling overview of the burden of visual impairment in Nigeria.
- Briefly acknowledging how cultural performing arts have historically influenced public health communication or social change in Nigeria would strengthen the theoretical and contextual rationale for using dance as an intervention modality.

**Response:** Agreed. We have added a brief acknowledgement of the historical role of performing arts (e.g., travelling theatre and community dance) in Nigerian health communication to provide a better context [Line 110-115].

2. Methods

(1) Study Design and Causal Interpretation: As this is a pre-post study without a control group, causal attribution is inherently limited.
- I recommend adopting consistently cautious language (e.g., “was associated with improvements”) rather than direct causal terms (e.g., “demonstrated effectiveness”) to accurately reflect the quasi-experimental nature of the study.

**Response:** We acknowledge the limitations of the quasi-experimental design. We have replaced direct causal language with more cautious terms such as "was associated with" or "suggests effectiveness" throughout the manuscript [See lines 410 – 411, Lines 32, 56, and 112].

(2) Sampling Strategy and Selection Bias: Participants were recruited from optometry clinics, suggesting they may already be more proactive about eye health.
- Please expand on how this selection bias may have contributed to the observed ceiling effects, particularly for outcomes related to health-seeking intention, and how it may have influenced effect size estimates.

**Response:** We have expanded the Discussion to clarify that recruiting from optometry clinics likely captured a "health-seeking" cohort. This baseline engagement explains the ceiling effect observed in the intention to visit a clinic (80% at baseline), which limited the intervention's observable impact on that specific metric [Lines 418-428]

(3) Statistical Significance Thresholds: The use of a significance threshold of p < 0.25 is highly unconventional for confirming public health outcomes.
- I encourage the authors to provide a brief but explicit justification for this choice (e.g., exploratory subgroup analyses). To enhance interpretability and comparability, reporting key findings using the conventional p < 0.05 threshold alongside the current results would be helpful.

**Response**: We appreciate the reviewer pointing this out. The threshold of *p*<0.25 was a typographical error and has been changed to the standard *p*<0.05.

3. Results

(1) Interpretation of Effect Sizes : Several outcomes achieve statistical significance but are associated with relatively modest effect sizes (e.g., Cramer’s V ≈ 0.07).
- Please clearly distinguish between statistical significance and practical public health relevance in the presentation and narrative interpretation of these results.

**Response:**

We thank the reviewer for this important statistical clarification. We have revised the Results and Discussion sections to explicitly distinguish between statistical significance and practical effect sizes. We acknowledge that a Cramer’s V of approximately 0.07 for overall knowledge gain indicates a modest effect size by standard statistical benchmarks.

However, we have added a narrative interpretation to the Discussion, highlighting the practical public health relevance of these findings. Specifically, we argue that for a low-cost, scalable digital intervention, even modest population-level shifts in knowledge are meaningful. Furthermore, we contextualise these modest overall effects as being suppressed by the 'ceiling effect' of our highly educated baseline sample. We contrast these modest general results with the substantial practical gains (e.g., knowledge jumps of over 60 percentage points) seen in the low-education subgroups, which represent the most significant contribution to health equity [Lines 440 – 449].

(2) Subgroup Analyses : The pronounced effects observed among participants with lower educational attainment represent a major strength of the study.
- Presenting these subgroup findings with greater visual or narrative prominence (e.g., figures, highlighted tables, or explicit comparison) would underscore the study’s contribution to health equity.

**Response:**

We thank the reviewer for this insightful observation. We agree that the most compelling evidence for the intervention’s effectiveness is found in its ability to close health literacy gaps among the most vulnerable subgroups. In the revised manuscript, we have elevated these findings to provide them with greater narrative and visual prominence. Specifically, we have restructured the Results section to explicitly contrast the high baseline awareness of participants with various levels of education. We have also expanded the Discussion to frame these results as a successful health equity strategy, demonstrating that culturally resonant visual media can transcend literacy barriers to empower underserved communities [Lines 483-488]

4. Discussion and Conclusion

(1) Sustainability and System Integration: The dance-integrated model is innovative and culturally resonant.
- Please discuss how this approach could be integrated into Nigeria’s primary healthcare or community health promotion frameworks to support long-term sustainability and scalability.

**Response**: We have added a section in the Discussion discussing how this can be integrated into the PHC[Lines 498-502]

(2) Cultural Social Capital : Participant engagement appears to be a key strength of the intervention.
- Framing dance as a form of cultural or social capital could provide a valuable theoretical lens to explain high engagement and message uptake, and would strengthen the conceptual contribution of the Discussion.

**Response:** We have added a section in the Discussion framing dance as a form of cultural social capital that enhances community trust. We also discuss integrating these videos into Nigeria’s Primary Healthcare (PHC) framework, perhaps via waiting-room screenings or community health worker toolkits[Lines 489-502]

(3) Long-Term Outcomes: The study primarily measures immediate post-intervention changes.
- In the Conclusion, please clearly distinguish between short-term attitudinal or intention-based changes and unmeasured long-term behavioural habits or clinical outcomes, and frame longer-term impacts as areas for future research.

**Response:** We have clarified in the Conclusion that the study measures immediate intention and knowledge gain rather than long-term behavioural habits, which remains a focus for future longitudinal research [Lines 543-544].

Reviewer #2: The authors have satisfactorily addressed the previous reviewer comments, and the manuscript has improved substantially in clarity and rigour.
The analyses are appropriate, the interpretation is suitably cautious, and the limitations are transparently discussed.
I believe the manuscript is suitable for publication, with only minor editorial refinements if deemed necessary by the editor.

**Response**: We thank you for the very useful comments.

7. PLOS authors have the option to publish the peer review history of their article ([what does this mean?](https://url.au.m.mimecastprotect.com/s/PqnkCE8wnyCWgDw91sPsQI77mhf?domain=track.editorialmanager.com)). If published, this will include your full peer review and any attached files.


**Do you want your identity to be public for this peer review?** If you choose “no”, your identity will remain anonymous but your review may still be made public.


For information about this choice, including consent withdrawal, please see our [Privacy Policy](https://url.au.m.mimecastprotect.com/s/L4zwCGv0pACJ0MrYWIktJIBKqCP?domain=track.editorialmanager.com).

Reviewer #1: No

Reviewer #2: **Yes:** Huiwon Jeon

[NOTE: If reviewer comments were submitted as an attachment file, they will be attached to this email and accessible via the submission site. Please log into your account, locate the manuscript record, and check for the action link "View Attachments". If this link does not appear, there are no attachment files.]

Figure Resubmissions:

While revising your submission, we strongly recommend that you use PLOS’s NAAS tool ([https://ngplosjournals.pagemajik.ai/artanalysis](https://url.au.m.mimecastprotect.com/s/e5z9CJyBv1c81YkrpiOuvIyr-uh?domain=ngplosjournals.pagemajik.ai)) to test your figure files. NAAS can convert your figure files to the TIFF file type and meet basic requirements (such as print size, resolution), or provide you with a report on issues that do not meet our requirements and that NAAS cannot fix.

After uploading your figures to PLOS’s NAAS tool - [https://ngplosjournals.pagemajik.ai/artanalysis](https://url.au.m.mimecastprotect.com/s/e5z9CJyBv1c81YkrpiOuvIyr-uh?domain=ngplosjournals.pagemajik.ai), NAAS will process the files provided and display the results in the "Uploaded Files" section of the page as the processing is complete. If the uploaded figures meet our requirements (or NAAS is able to fix the files to meet our requirements), the figure will be marked as "fixed" above. If NAAS is unable to fix the files, a red "failed" label will appear above. When NAAS has confirmed that the figure files meet our requirements, please download the file via the download option, and include these NAAS processed figure files when submitting your revised manuscript.

**Response: Done**
