# Supplementary material for: Feasibility and immediate effects of a multimodal, dance-integrated educational video package on eye health knowledge and intentions in Nigeria: A pre-post pilot study
Source: PLOS Glob Public Health. 2026 Oct 14;6(10):e0005585. doi: 10.1371/journal.pgph.0005585 (PMC13667777; doi:10.1371/journal.pgph.0005585)
Supplement: Supplementary file 6 [file pgph.0005585.s008.docx]

Dear Editor

On behalf of the authors, I wish to sincerely thank you for helping to improve this study and make it a very strong paper. We truly appreciate it.

Below we have tabulated the responses indicating the resolutions and changes made for each of the suggested revisions. In the main manuscript, these changes were highlighted in the tracked version for easy identification.

We hope this will be accepted by your journal.

| Reviewers comment | Response to comments as inscribed in the manuscripts |
| --- | --- |
| Reviewer 3 |  |
| In line 98, where the authors state “the intervention not only increases awareness…”, it would be helpful for the authors to restate/clarify the intervention being referred to. At present, the reader is required to infer this from the preceding description, which may reduce clarity. | By integrating these concepts, the dance -integrated educational videos not only increases awareness but also boosts participation and promotes the adoption of preventive eye health behaviours.(line 99) |
|  |  |
| Line 167 - 176: The description of the intervention is clear in terms of video production and delivery. However, it would be helpful for the authors to clarify how the educational content (e.g., symptoms and information on myopia, cataracts, glaucoma, and general eye health) within the videos were developed. Were these messages developed by the research team, adapted from existing guidelines or public health resources? | The educational content incorporated into the videos was developed by the research team, comprising optometrists and eye health researchers, based on current evidence and established eye health guidelines. Key messages on the symptoms, risk factors, prevention, and management of myopia, cataracts, glaucoma, and general eye health were adapted from internationally recognised eye health resources and the existing scientific literature. The content was reviewed by experienced optometrists to ensure scientific accuracy, clinical relevance, and suitability for the Nigerian context before video production(line 172 to 179) |
|  |  |
| Line 349: There appears to be a grammatical error in this sentence due to repetition (“the impact of videos” is duplicated). Kindly check to amend it. | Well noted and rectify in the manuscript line 348 |
| Reviewer 4 |  |
| However, there is a fundamental misalignment among the study design, the terminology used (including “quasi-experimental” and “effectiveness”), and the level of causal inference claimed in the manuscript. | Association (line 1); showed positive changes. (line 49); positive changes (line 57); associated with positive changes in (line 535).  We also Replaced "effectiveness" with "impact" in these sections. "This study evaluated the impact of dance-integrated educational videos..."  "The impact of the interventions would be better demonstrated by.. |
|  |  |
| The study should be consistently described as a pre–post educational exposure study (non-controlled quasi-experimental or pilot intervention study), not an effectiveness evaluation. | This was a quantitative pre and post intervention educational exposure study(line 125). |
|  |  |
| Behavioural outcomes should be clearly distinguished as self-reported intentions or immediate responses, not behavioral adoption. • Claims of behaviour change should be moderated accordingly | line(s) Current text Suggested revision  48–50 "Self-reported intentions to adopt protective eye health practices, such as wearing sunglasses, reducing screen time, and scheduling regular eye examinations, increased immediately following exposure to the educational videos."  386–387 ""Participants reported significantly greater intentions to adopt protective eye health practices immediately following exposure to the educational videos."  474-475 "participants reported stronger intentions and greater willingness to engage in preventive eye health practices immediately after viewing the educational videos."  542–544 (Conclusion) "...was associated with improvements in knowledge, perceived understanding, and immediate self-reported intentions to engage in preventive eye health practices following exposure to the educational videos. |
|  |  |
| There is a disconnect between the acknowledged limitations (which are appropriate and strong) and the interpretation in the discussion/conclusion (which remains overly strong). This inconsistency must be resolved for scientific coherence. | Thanks for this observation. We have softened the adjectives in the Discussion section to reflect the "pilot" nature of the work.  Where we used strong terms like "most profound impact" and "bridged massive literacy gaps, we changed to "most notable impact" and "reduced substantial literacy gaps.". This makes the tone more objective and less promotional  The below as also included in the study: This study serves as a valuable pilot, demonstrating the feasibility and acceptability of this innovative .(line 552-553) |
| The manuscript’s value lies in the feasibility of the intervention, the acceptability of culturally tailored video delivery, and preliminary evidence of short-term knowledge gain. It should therefore be positioned as a pilot or exploratory intervention study, not a demonstration of effectiveness. | Thank you for this observation. The manuscript tone and study design have now been revised inline with this suggestion. See previous responses above |
|  |  |
| Reviewer 5 |  |
| I thank the authors for their careful and constructive revision of the manuscript. The revised version has addressed the comments raised in the previous round of review, and the manuscript is now clearer, better contextualised, and more appropriately framed in relation to its quasi experimental design. The distinction between health education and health promotion has been strengthened, and the role of dance as a culturally resonant mechanism for engagement, recall, and behavioural motivation is now more clearly explained.  The methodology is sufficiently described, including the recruitment strategy, intervention format, data collection procedures, questionnaire development, pilot testing, reliability assessment, ethical approval, and statistical analysis. The authors have also adopted more cautious language when interpreting the findings, which is appropriate given the absence of a control group and the immediate post intervention measurement of outcomes. The conclusions are broadly supported by the data presented, particularly the improvements in eye health knowledge, perceived understanding, protective behaviours, and the strong response among participants with lower educational attainment.  The statistical analysis appears appropriate for the study design and type of data. The use of paired comparisons, McNemar’s tests for categorical pre and post comparisons, and the acknowledgement of multiple subgroup analyses and Bonferroni correction strengthen the analysis. The limitations are also now adequately recognised, including selection bias from clinic based recruitment, self reported outcomes, absence of long term follow up, lack of a control group, and the risk of Type 1 error in subgroup analyses.  Overall, this is an innovative and culturally relevant public health study with potential value for scalable eye health promotion in Nigeria and similar resource limited settings. I have no further substantive concerns regarding the manuscript, the ethical conduct of the study, data availability, or publication ethics. Subject to any final editorial checks, I consider the manuscript acceptable for publication.  Response: Thank you for the kind messages and for helping to strengthen this manuscript. | |
|  |  |
