# Supplementary material for: Feasibility and immediate effects of a multimodal, dance-integrated educational video package on eye health knowledge and intentions in Nigeria: A pre-post pilot study
Source: PLOS Glob Public Health. 2026 Oct 14;6(10):e0005585. doi: 10.1371/journal.pgph.0005585 (PMC13667777; doi:10.1371/journal.pgph.0005585)
Supplement: Supplementary file 7 [file pgph.0005585.s009.docx]

| Reviewers comment | Response to comments as inscribed in the manuscripts |
| --- | --- |
| 1. Please reconsider: (i) the terminology used to describe the intervention and ensure that the title, abstract, introduction, and conclusions accurately reflect what was actually tested. (ii) any conclusions regarding the educational effectiveness of dance and these should be substantially tempered, as the current study design does not isolate the contribution of the dance component. | We completely agree with this critical methodological point. Because our study evaluated a composite intervention (factual eye-health education combined with choreographed dance) without an active, standard-education control group, we cannot statistically isolate the independent causal contribution of the dance choreography alone.  To address this the following changes were made:  1. We have revised the terminology throughout the Title, Abstract, Methods, and Discussion to consistently frame the intervention as a "multimodal, dance-integrated educational video package" or "composite art-based health communication tool."  2. We have tempered our claims throughout, changing definitive causal language to association-based and pilot-level interpretations (e.g., "associated with immediate post-exposure intentions").  3. We have explicitly added this design limitation to the *Limitations and Strengths* section and outlined the need for future three-arm randomized trials to isolate the specific effect sizes of the visual, text, and performative components. |
| 2. Given the participants were recruited from clinics, how do the authors interpret the finding that “while 168 (38.4%) had never attended an eye clinic” on page 12, LN 248-251 | The participating clinics served primarily as designated meeting points for study participation and data collection, rather than as the primary source of recruitment. Participants were recruited from community outreaches and were invited to the clinic if they indicated interest. This is how the proportion of nonattendance to eye clinic was captured. This recruitment strategy has been clearly explained in the methods. see line 134 to 150. |
| The purpose of the chi-squared (χ²) tests reported in Table 1 is unclear. Please specify the comparison or hypothesis being tested. | We have removed the chi-square text and Table 1 now serves strictly as a descriptive demographic baseline. Running hypothesis testing on demographic baseline totals without a clear a priori clinical hypothesis can lead to Type 1 error inflation. Thanks for this observation. |
| 3.On page 12, LN 252-255, the authors stated that “Among participants who reported being diagnosed with an eye condition, the most common diagnoses were myopia, presbyopia, astigmatism, and hyperopia (Figure 1)”. Could the authors clarify how the data presented in Figure 1 were obtained and categorised? Several of the reported responses (e.g., astigmatism, conjunctivitis, and ocular superinfection) are technical diagnostic terms. Please indicate whether participants generated these terms spontaneously in response to open-ended questions, selected them from predefined response options, or whether participants' descriptions were subsequently coded by the research team into diagnostic categories. Additional detail on this process would aid interpretation of the findings. | We thank the reviewer for this important clarification. The eye conditions presented in Figure 1 were obtained directly from a structured questionnaire item asking participants whether they had ever been diagnosed with an eye condition, with response options of “Yes” or “No.” Participants who answered “Yes” were then asked to specify the eye condition from a list of predefined diagnostic categories provided in the questionnaire.  Importantly, reporting a previous eye condition was not an eligibility criterion and did not affect participants’ ability to participate in the educational video intervention. The diagnostic terms presented in Figure 1 were therefore not spontaneously generated by participants in response to an open-ended question, nor were participants’ descriptions subsequently coded into diagnostic categories by the research team. Rather, they represent participants’ selections from the predefined diagnostic categories in the questionnaire.  We have clarified this process in the revised Methods section and Figure 1 description to ensure that the source and categorisation of these data are transparent. |
| 4.Page 12, LN 258-266 and Page 15, LN 308-324, it’s not clear which table or figure the results originate from. While the text interprets the statistical findings, there is no accompanying table or figure presenting the underlying data. | Sorry for the omission. The table has been inserted for better clarification. Table 2 to 3 (line 332 -339) and table 4 and 5 (see line 336 -337;355-368). |
|  |  |
| 5. The intervention videos are currently provided via Google Drive links in the supplementary material. Could the authors consider depositing the videos in a permanent, citable repository (e.g., Zenodo, Figshare, OSF) to ensure that the intervention remains available to readers, facilitate reproducibility, and allow future researchers to evaluate and replicate the intervention? | We thank the reviewer for this excellent suggestion, which greatly enhances the reproducibility and transparency of our work. We have deposited all four intervention videos in a permanent, open-access, and publicly citable repository on **Figshare**. DOI: 10.6084/m9.figshare.33227856 |
|  |  |
