# Supplementary material for: Feasibility and immediate effects of a multimodal, dance-integrated educational video package on eye health knowledge and intentions in Nigeria: A pre-post pilot study
Source: PLOS Glob Public Health. 2026 Oct 14;6(10):e0005585. doi: 10.1371/journal.pgph.0005585 (PMC13667777; doi:10.1371/journal.pgph.0005585)
Supplement: Supplementary file 8 [file pgph.0005585.s010.docx]

**Response to Editor/Reviewer Comments**

Journal: *PLOS ONE* Manuscript Title: *Feasibility and Immediate Effects of a Multimodal, Dance-Integrated Educational Video Package on Eye Health Knowledge and Intentions in Nigeria: A Pre-Post Pilot Study* Manuscript ID: PGPH-D-25-03403R4

Dear Editor and Reviewers,

We sincerely thank the Academic Editor and the peer reviewers for their thoughtful, constructive, and thorough evaluation of our manuscript. Their valuable feedback and insightful suggestions have significantly strengthened the rigor, methodological clarity, and overall impact of our paper.

In addition to addressing all specific points raised during the review, we have conducted a comprehensive review and revision of the manuscript to enhance language, tone, sentence structure, and overall comprehension throughout all sections. Causal language has been systematically moderated to reflect the quasi-experimental design, statistical reporting has been further clarified, and additional context has been integrated into the Introduction and Discussion sections.

Below, we provide a point-by-point response detailing how each comment has been addressed, along with exact location references in the newly revised manuscript.

Point-by-Point Response Table

| **#** | **Reviewer / Editor Comment** | **Author Response & Action Taken** | **Location in Revised Manuscript** |
| --- | --- | --- | --- |
| 1 | Title and Abstract Redundancy: Simplify redundant phrasing such as "pre–post interventional pilot intervention study" in the title and abstract. | Agreed. We have simplified the title and abstract text to eliminate redundant terminology. The title now reads "...A Pre-Post Pilot Study" and the Abstract Methods section reflects "a pre–post pilot intervention study." | Title Page [line 1-3]; Abstract (Methods) [line 34.] |
| 2 | Rationale for Dance & English Literacy Requirement: Reconcile the claim that performing arts reach audiences "regardless of literacy level" with the study's inclusion requirement for English literacy. Clarify the specific engagement mechanism of the dance package. | Agreed and clarified. We removed the "regardless of literacy level" justification since our participants were required to read and write English. Instead, we re-framed the core rationale around audience engagement and attention retention—explaining that while lengthy text-based health materials easily lead to disengagement, dance-integrated videos capture attention, foster emotional engagement, and encourage retention. Furthermore, we added a theoretical distinction between *health education* (factual messaging) and *health promotion* (dance-based engagement). | Introduction line 117 -125. |
| 3 | Disaggregation of Age Groupings: Justify or disaggregate the broad "18–44 young adults" grouping to ensure age-specific trends are visible. | Addressed. We clarified in the text that the 18–44 range was collapsed into a "young adults" umbrella term solely for high-level descriptive summary. Crucially, the more granular, standardized age bands (18–24, 25–34, 35–44, 45–54, 55+) are fully disaggregated in Table 1 and were retained for all subgroup cross-tabulations and Chi-square analyses. | Results (Participant Demographics) Table 1 see line 274-275 see also line 265-269 |
| 4 | Sparse Expected Cell Counts (Exact Testing): Verify contingency table Chi-square analyses for Tables 3 and 5 where sparse cell counts (<5) were observed. | Completed. To account for sparse cell counts ($<5$ expected counts in $>20\%$ of cells), we computed Monte Carlo exact Fisher–Freeman–Halton test $p$-values based on 200,000 sampled tables. The exact tests confirmed the asymptotic Chi-square results across all demographic categories. Footnotes and narrative text have been updated accordingly. | Methods (Data management and Analysis) [lines 249-252]; Table 3 ( line 354) Table 5 Footnotes [see lines 381] |
| 5 | Moderation of Causal Language: Soften definitive causal verbs (e.g., "led to", "effect of") to align with the quasi-experimental, pre-post single-arm pilot study design. | Agreed and updated. We systematically audited the entire manuscript and replaced causal phrasing with appropriate associative terms (e.g., "associated with", "pre- and post-exposure differences", "changes in intention"). Captions, subheadings, and narrative text in the Abstract, Results, and Discussion have been modified. | Abstract [line 34]; Results & Captions; [Line 322, 323, 342, 351,352, 367, 578 etc] |
| 6 | Self-Reported Diagnostic Terms Comprehension: Address potential misclassification or under-reporting of technical ocular terms (e.g., astigmatism, presbyopia) among lower-education participants. | Addressed. We added an explicit explanation detailing how the questionnaire terms were presented and administered with research assistant support. Furthermore, we explicitly acknowledged the potential for under-reporting or misclassification of self-reported clinical diagnoses among lower-literacy subgroups as a study limitation. | Methods (Data Collection) [line 214 -224]; Limitations [lines 553-557] |
| 7 | Recruitment Strategy & Selection Bias: Clarify the recruitment venue and address selection bias regarding clinic-based sampling versus community outreach. | Clarified. We expanded the Methods section to explain that recruitment was initiated during community outreach activities, with local optometry clinics designated as safe, equipped meeting hubs for questionnaire completion and video viewing. We also discussed how clinic-hub data collection captured a health-seeking sample, contributing to a baseline ceiling effect in clinic attendance intention. | Methods (Recruitment) [line 142-162]. |
| 8 | Language, Grammar, and Overall Comprehension: Ensure the manuscript is thoroughly proofread for grammar, sentence flow, typographical consistency, and scientific clarity. | Completed. The manuscript underwent comprehensive editorial revision. Sentence fragments were repaired, mathematical terminology was corrected (distinguishing absolute percentage-point changes from relative percentage increases), formatting of sample size formulas was cleaned up, and overall readability was significantly enhanced. | Across the manuscript |
