# Supplementary material for: Feasibility and immediate effects of a multimodal, dance-integrated educational video package on eye health knowledge and intentions in Nigeria: A pre-post pilot study
Source: PLOS Glob Public Health. 2026 Oct 14;6(10):e0005585. doi: 10.1371/journal.pgph.0005585 (PMC13667777; doi:10.1371/journal.pgph.0005585)
Supplement: Peer review history

# Editor report: Decision Letter 0

**DOI:** 10.1371/journal.pgph.0005585.r001

**Authors:** Hocheol Lee

2 Dec 2025

PGPH-D-25-03403

Effectiveness of Dance-Integrated Educational Videos in Improving Eye Health Knowledge and Preventive Behaviours in Nigeria

PLOS Global Public Health

Dear Dr. Osuagwu,

Thank you for submitting your manuscript to PLOS Global Public Health. After careful consideration, we feel that it has merit but does not fully meet PLOS Global Public Health’s publication criteria as it currently stands. Therefore, we invite you to submit a revised version of the manuscript that addresses the points raised during the review process.

Please submit your revised manuscript by Jan 16 2026 11:59PM. If you will need more time than this to complete your revisions, please reply to this message or contact the journal office at globalpubhealth@plos.org. When you're ready to submit your revision, log on to https://www.editorialmanager.com/pgph/ and select the 'Submissions Needing Revision' folder to locate your manuscript file.

Please include the following items when submitting your revised manuscript:

- A rebuttal letter that responds to each point raised by the editor and reviewer(s). You should upload this letter as a separate file labeled 'Response to Reviewers'.
- A marked-up copy of your manuscript that highlights changes made to the original version. You should upload this as a separate file labeled 'Revised Manuscript with Track Changes'.
- An unmarked version of your revised paper without tracked changes. You should upload this as a separate file labeled 'Manuscript'.

Guidelines for resubmitting your figure files are available below the reviewer comments at the end of this letter.

We look forward to receiving your revised manuscript.

Kind regards,

Hocheol Lee

Academic Editor

PLOS Global Public Health

Journal Requirements:

If the reviewer comments include a recommendation to cite specific previously published works, please review and evaluate these publications to determine whether they are relevant and should be cited. There is no requirement to cite these works unless the editor has indicated otherwise.

Additional Editor Comments (if provided):

'The manuscript titled ‘Effectiveness of Dance-Integrated Educational Videos in Improving Eye Health Knowledge and Preventive Behaviours in Nigeria’ presents important and timely evidence on eye health in Nigeria.

However, several areas require further refinement, and a subsequent round of review appears necessary.

[Note: HTML markup is below. Please do not edit.]

Reviewers' comments:

[NOTE: If reviewer comments were submitted as an attachment file, they will be attached to this email and accessible via the submission site. Please log into your account, locate the manuscript record, and check for the action link "View Attachments". If this link does not appear, there are no attachment files.]

# Author comment: Author response to Decision Letter 1

**DOI:** 10.1371/journal.pgph.0005585.r002

24 Dec 2025

Attachment Submitted filename: Response to Reviewers dance updated.docx

# Peer review: Decision Letter 1

**DOI:** 10.1371/journal.pgph.0005585.r003

**Authors:** Hocheol Lee

23 Feb 2026

PGPH-D-25-03403R1

Effectiveness of Dance-Integrated Educational Videos in Improving Eye Health Knowledge and Preventive Behaviours in Nigeria

PLOS Global Public Health

Dear Dr. Osuagwu,

Thank you for submitting your manuscript to PLOS Global Public Health. After careful consideration, we feel that it has merit but does not fully meet PLOS Global Public Health’s publication criteria as it currently stands. Therefore, we invite you to submit a revised version of the manuscript that addresses the points raised during the review process.

Please submit your revised manuscript by Mar 25 2026 11:59PM. If you will need more time than this to complete your revisions, please reply to this message or contact the journal office at globalpubhealth@plos.org. When you're ready to submit your revision, log on to https://www.editorialmanager.com/pgph/ and select the 'Submissions Needing Revision' folder to locate your manuscript file.

Please include the following items when submitting your revised manuscript:

- A letter that responds to each point raised by the editor and reviewer(s). You should upload this letter as a separate file labeled 'Response to Reviewers'.
- A marked-up copy of your manuscript that highlights changes made to the original version. You should upload this as a separate file labeled 'Revised Manuscript with Track Changes'.
- An unmarked version of your revised paper without tracked changes. You should upload this as a separate file labeled 'Manuscript'.

Guidelines for resubmitting your figure files are available below the reviewer comments at the end of this letter.

We look forward to receiving your revised manuscript.

Kind regards,

Hocheol Lee

Academic Editor

PLOS Global Public Health

Journal Requirements:

If the reviewer comments include a recommendation to cite specific previously published works, please review and evaluate these publications to determine whether they are relevant and should be cited. There is no requirement to cite these works unless the editor has indicated otherwise.

Please review your reference list to ensure that it is complete and correct. If you have cited papers that have been retracted, please include the rationale for doing so in the manuscript text, or remove these references and replace them with relevant current references. Any changes to the reference list should be mentioned in the rebuttal letter that accompanies your revised manuscript. If you need to cite a retracted article, indicate the article’s retracted status in the References list and also include a citation and full reference for the retraction notice.

Additional Editor Comments (if provided):

The Editorial Office sincerely thanks the authors for their careful and thorough revisions across two rounds of review.

After consideration of the reviewers’ comments, we invite the authors to revise the manuscript accordingly, taking into full account the points raised by both reviewers. We kindly ask that a detailed, point-by-point response to each comment be provided upon resubmission.

We look forward to receiving your revised manuscript.

[Note: HTML markup is below. Please do not edit.]

Reviewers' comments:

Reviewer's Responses to Questions

**Comments to the Author**

Reviewer #1: (No Response)

Reviewer #2: All comments have been addressed

**********

publication criteria?

Reviewer #1: (No Response)

Reviewer #2: Yes

**********

3. Has the statistical analysis been performed appropriately and rigorously?

Reviewer #1: (No Response)

Reviewer #2: Yes

**********

4. Have the authors made all data underlying the findings in their manuscript fully available (please refer to the Data Availability Statement at the start of the manuscript PDF file)?

The PLOS Data policy

Reviewer #1: (No Response)

Reviewer #2: Yes

**********

5. Is the manuscript presented in an intelligible fashion and written in standard English?

Reviewer #1: (No Response)

Reviewer #2: Yes

**********

Reviewer #1: I would like to commend the authors for their creative and culturally resonant approach to eye health promotion in Nigeria. Utilizing dance-integrated videos is an innovative way to bridge the gap in health literacy, particularly in resource-limited settings. The finding that this intervention was especially effective among participants with lower educational attainment is highly encouraging from a public health equity perspective. While the study has clear merits, I believe addressing the following points organized by section would further strengthen the manuscript.

1. Introduction

(1) Conceptual Framework: The manuscript distinguishes between health education and health promotion.

- Please elaborate on how the dance component functions specifically as a health promotion mechanism, particularly in motivating behavioural change beyond simple information delivery (e.g., emotional engagement, social modeling, cultural resonance).

(2) Contextual Background: The authors provide a compelling overview of the burden of visual impairment in Nigeria.

- Briefly acknowledging how cultural performing arts have historically influenced public health communication or social change in Nigeria would strengthen the theoretical and contextual rationale for using dance as an intervention modality.

2. Methods

(1) Study Design and Causal Interpretation: As this is a pre-post study without a control group, causal attribution is inherently limited.

- I recommend adopting consistently cautious language (e.g., “was associated with improvements”) rather than direct causal terms (e.g., “demonstrated effectiveness”) to accurately reflect the quasi-experimental nature of the study.

(2) Sampling Strategy and Selection Bias: Participants were recruited from optometry clinics, suggesting they may already be more proactive about eye health.

- Please expand on how this selection bias may have contributed to the observed ceiling effects, particularly for outcomes related to health-seeking intention, and how it may have influenced effect size estimates.

(3) Statistical Significance Thresholds : The use of a significance threshold of p < 0.25 is highly unconventional for confirming public health outcomes.

- I encourage the authors to provide a brief but explicit justification for this choice (e.g., exploratory subgroup analyses). To enhance interpretability and comparability, reporting key findings using the conventional p < 0.05 threshold alongside the current results would be helpful.

3. Results

(1) Interpretation of Effect Sizes : Several outcomes achieve statistical significance but are associated with relatively modest effect sizes (e.g., Cramer’s V ≈ 0.07).

- Please clearly distinguish between statistical significance and practical public health relevance in the presentation and narrative interpretation of these results.

(2) Subgroup Analyses : The pronounced effects observed among participants with lower educational attainment represent a major strength of the study.

- Presenting these subgroup findings with greater visual or narrative prominence (e.g., figures, highlighted tables, or explicit comparison) would underscore the study’s contribution to health equity.

4. Discussion and Conclusion

(1) Sustainability and System Integration : The dance-integrated model is innovative and culturally resonant.

- Please discuss how this approach could be integrated into Nigeria’s primary healthcare or community health promotion frameworks to support long-term sustainability and scalability.

(2) Cultural Social Capital : Participant engagement appears to be a key strength of the intervention.

- Framing dance as a form of cultural or social capital could provide a valuable theoretical lens to explain high engagement and message uptake, and would strengthen the conceptual contribution of the Discussion.

(3) Long-Term Outcomes : The study primarily measures immediate post-intervention changes.

- In the Conclusion, please clearly distinguish between short-term attitudinal or intention-based changes and unmeasured long-term behavioural habits or clinical outcomes, and frame longer-term impacts as areas for future research.

Reviewer #2: The authors have satisfactorily addressed the previous reviewer comments, and the manuscript has improved substantially in clarity and rigor.

The analyses are appropriate, the interpretation is suitably cautious, and the limitations are transparently discussed.

I believe the manuscript is suitable for publication, with only minor editorial refinements if deemed necessary by the editor.

**********

what does this mean?). If published, this will include your full peer review and any attached files.

**Do you want your identity to be public for this peer review?**  If you choose “no”, your identity will remain anonymous but your review may still be made public.

For information about this choice, including consent withdrawal, please see our Privacy Policy

Reviewer #1: No

Reviewer #2: **Yes:**  Huiwon Jeon

**********

[NOTE: If reviewer comments were submitted as an attachment file, they will be attached to this email and accessible via the submission site. Please log into your account, locate the manuscript record, and check for the action link "View Attachments". If this link does not appear, there are no attachment files.]

# Author comment: Author response to Decision Letter 2

**DOI:** 10.1371/journal.pgph.0005585.r004

6 Mar 2026

Attachment Submitted filename: Review comments24022026.docx

# Peer review: Decision Letter 2

**DOI:** 10.1371/journal.pgph.0005585.r005

**Authors:** Emmanuel Olamijuwon

23 Jun 2026

PGPH-D-25-03403R2

Effectiveness of Dance-Integrated Educational Videos in Improving Eye Health Knowledge and Preventive Behaviours in Nigeria

PLOS Global Public Health

Dear Dr. Osuagwu,

Thank you for submitting your manuscript to PLOS Global Public Health. After careful consideration, we feel that it has merit but does not fully meet PLOS Global Public Health’s publication criteria as it currently stands. Therefore, we invite you to submit a revised version of the manuscript that addresses the points raised during the review process.

Please submit your revised manuscript by Jul 23 2026 11:59PM. If you will need more time than this to complete your revisions, please reply to this message or contact the journal office at globalpubhealth@plos.org. When you're ready to submit your revision, log on to https://www.editorialmanager.com/pgph/ and select the 'Submissions Needing Revision' folder to locate your manuscript file.

Please include the following items when submitting your revised manuscript:

- A letter that responds to each point raised by the editor and reviewer(s). You should upload this letter as a separate file labeled 'Response to Reviewers'.
- A marked-up copy of your manuscript that highlights changes made to the original version. You should upload this as a separate file labeled 'Revised Manuscript with Track Changes'.
- An unmarked version of your revised paper without tracked changes. You should upload this as a separate file labeled 'Manuscript'.

Guidelines for resubmitting your figure files are available below the reviewer comments at the end of this letter.

As the corresponding author, your ORCID iD is verified in the submission system and will appear in the published article. PLOS supports the use of ORCID, and we encourage all coauthors to register for an ORCID iD and use it as well. Please encourage your coauthors to verify their ORCID iD within the submission system before final acceptance, as unverified ORCID iDs will not appear in the published article. *Only*  the individual author can complete the verification step; PLOS staff *cannot*  verify ORCID iDs on behalf of authors.

We look forward to receiving your revised manuscript.

Kind regards,

Emmanuel Olamijuwon, Ph.D

Academic Editor

PLOS Global Public Health

Journal Requirements:

If the reviewer comments include a recommendation to cite specific previously published works, please review and evaluate these publications to determine whether they are relevant and should be cited. There is no requirement to cite these works unless the editor has indicated otherwise.

Please review your reference list to ensure that it is complete and correct. If you have cited papers that have been retracted, please include the rationale for doing so in the manuscript text, or remove these references and replace them with relevant current references. Any changes to the reference list should be mentioned in the rebuttal letter that accompanies your revised manuscript. If you need to cite a retracted article, indicate the article’s retracted status in the References list and also include a citation and full reference for the retraction notice.

Additional Editor Comments (if provided):

[Note: HTML markup is below. Please do not edit.]

Reviewers' comments:

Reviewer's Responses to Questions

**Comments to the Author**

Reviewer #3: (No Response)

Reviewer #4: (No Response)

Reviewer #5: All comments have been addressed

**********

publication criteria?

Reviewer #3: Yes

Reviewer #4: No

Reviewer #5: Yes

**********

3. Has the statistical analysis been performed appropriately and rigorously?

Reviewer #3: Yes

Reviewer #4: No

Reviewer #5: Yes

**********

4. Have the authors made all data underlying the findings in their manuscript fully available (please refer to the Data Availability Statement at the start of the manuscript PDF file)?

The PLOS Data policy

Reviewer #3: Yes

Reviewer #4: Yes

Reviewer #5: Yes

**********

5. Is the manuscript presented in an intelligible fashion and written in standard English?

Reviewer #3: Yes

Reviewer #4: Yes

Reviewer #5: Yes

**********

Reviewer #3: This is a very good study that uses an innovative and creative approach to address eye health and improve the knowledge, awareness, and behaviours of people, particularly in Nigeria. There are, however, a few comments that the authors should consider.

- In line 98, where the authors state “the intervention not only increases awareness…”, it would be helpful for the authors to restate/clarify the intervention being referred to. At present, the reader is required to infer this from the preceding description, which may reduce clarity.

- Line 167 - 176: The description of the intervention is clear in terms of video production and delivery. However, it would be helpful for the authors to clarify how the educational content (e.g., symptoms and information on myopia, cataracts, glaucoma, and general eye health) within the videos were developed. Were these messages developed by the research team, adapted from existing guidelines or public health resources?

- Line 349: There appears to be a grammatical error in this sentence due to repetition (“the impact of videos” is duplicated). Kindly check to amend it.

Reviewer #4: The manuscript presents an innovative intervention that uses dance-integrated educational videos to promote knowledge of eye health and preventive intentions in Nigeria. The study addresses an important gap in health communication strategies in low-resource and low-literacy settings, and the dataset is substantial. However, there is a fundamental misalignment among the study design, the terminology used (including “quasi-experimental” and “effectiveness”), and the level of causal inference claimed in the manuscript.

The study is effectively a single-group, immediate pre-post exposure assessment without a control group, and self-reported immediate outcomes following an exposure to one or multiple three-minute videos. While this design can provide useful insights into short-term changes following exposure, it does not support causal inference or claims of intervention effectiveness.

Given that the authors have also acknowledged key limitations, including the absence of a control group, selection bias, self-reported outcomes, and a lack of longitudinal follow-up, the current framing of the manuscript appears inconsistent with these acknowledged constraints.

The manuscript requires major reframing, specifically:

1. Alignment of claims with design

• Remove or soften causal language such as “effectiveness,” “improved,” and “achieved impact.”

• Replace with “associated with,” “following exposure,” or “short-term changes observed after intervention exposure."

2. Reclassification of study design

The study should be consistently described as a pre–post educational exposure study (non-controlled quasi-experimental or pilot intervention study), not an effectiveness evaluation.

3. Interpretation of outcomes

• Behavioural outcomes should be clearly distinguished as self-reported intentions or immediate responses, not behavioral adoption.

• Claims of behaviour change should be moderated accordingly

4. Internal consistency: There is a disconnect between the acknowledged limitations (which are appropriate and strong) and the interpretation in the discussion/conclusion (which remains overly strong).

This inconsistency must be resolved for scientific coherence.

The manuscript’s value lies in the feasibility of the intervention, the acceptability of culturally tailored video delivery,

and preliminary evidence of short-term knowledge gain. It should therefore be positioned as a pilot or exploratory intervention study, not a demonstration of effectiveness.

Reviewer #5: I thank the authors for their careful and constructive revision of the manuscript. The revised version has addressed the comments raised in the previous round of review, and the manuscript is now clearer, better contextualised, and more appropriately framed in relation to its quasi experimental design. The distinction between health education and health promotion has been strengthened, and the role of dance as a culturally resonant mechanism for engagement, recall, and behavioural motivation is now more clearly explained.

The methodology is sufficiently described, including the recruitment strategy, intervention format, data collection procedures, questionnaire development, pilot testing, reliability assessment, ethical approval, and statistical analysis. The authors have also adopted more cautious language when interpreting the findings, which is appropriate given the absence of a control group and the immediate post intervention measurement of outcomes. The conclusions are broadly supported by the data presented, particularly the improvements in eye health knowledge, perceived understanding, protective behaviours, and the strong response among participants with lower educational attainment.

The statistical analysis appears appropriate for the study design and type of data. The use of paired comparisons, McNemar’s tests for categorical pre and post comparisons, and the acknowledgement of multiple subgroup analyses and Bonferroni correction strengthen the analysis. The limitations are also now adequately recognised, including selection bias from clinic based recruitment, self reported outcomes, absence of long term follow up, lack of a control group, and the risk of Type 1 error in subgroup analyses.

Overall, this is an innovative and culturally relevant public health study with potential value for scalable eye health promotion in Nigeria and similar resource limited settings. I have no further substantive concerns regarding the manuscript, the ethical conduct of the study, data availability, or publication ethics. Subject to any final editorial checks, I consider the manuscript acceptable for publication.

**********

what does this mean?). If published, this will include your full peer review and any attached files.

**Do you want your identity to be public for this peer review?**  If you choose “no”, your identity will remain anonymous but your review may still be made public.

For information about this choice, including consent withdrawal, please see our Privacy Policy

Reviewer #3: No

Reviewer #4: **Yes:**  Grace Ibor

Reviewer #5: **Yes:**  Ishrat Islam

**********

[NOTE: If reviewer comments were submitted as an attachment file, they will be attached to this email and accessible via the submission site. Please log into your account, locate the manuscript record, and check for the action link "View Attachments". If this link does not appear, there are no attachment files.]

# Author comment: Author response to Decision Letter 3

**DOI:** 10.1371/journal.pgph.0005585.r006

4 Jul 2026

Attachment Submitted filename: Response to reviewerscommentsv2.docx

# Editor report: Decision Letter 3

**DOI:** 10.1371/journal.pgph.0005585.r007

**Authors:** Emmanuel Olamijuwon

19 Jul 2026

PGPH-D-25-03403R3

Association of Dance-Integrated Educational Videos in Improving Eye Health Knowledge and Preventive Behaviours in Nigeria

PLOS Global Public Health

Dear Dr. Osuagwu,

Thank you for submitting your manuscript to PLOS Global Public Health. After careful consideration, we feel that it has merit but does not fully meet PLOS Global Public Health’s publication criteria as it currently stands. Therefore, we invite you to submit a revised version of the manuscript that addresses the points raised during the review process.

Before the manuscript is sent for further peer review, please address the editorial comments listed below. Your responses should be incorporated into the existing point-by-point response to the reviewers' comments, with a clear indication of how each issue has been addressed in the revised manuscript. The revised manuscript and consolidated response document will then be considered for further peer review.

Please submit your revised manuscript by Sep 17 2026 11:59PM. If you will need more time than this to complete your revisions, please reply to this message or contact the journal office at globalpubhealth@plos.org. When you're ready to submit your revision, log on to https://www.editorialmanager.com/pgph/ and select the 'Submissions Needing Revision' folder to locate your manuscript file.

Please include the following items when submitting your revised manuscript:

- A letter that responds to each point raised by the editor and reviewer(s). You should upload this letter as a separate file labeled 'Response to Reviewers'.
- A marked-up copy of your manuscript that highlights changes made to the original version. You should upload this as a separate file labeled 'Revised Manuscript with Track Changes'.
- An unmarked version of your revised paper without tracked changes. You should upload this as a separate file labeled 'Manuscript'.

Guidelines for resubmitting your figure files are available below the reviewer comments at the end of this letter.

As the corresponding author, your ORCID iD is verified in the submission system and will appear in the published article. PLOS supports the use of ORCID, and we encourage all coauthors to register for an ORCID iD and use it as well. Please encourage your coauthors to verify their ORCID iD within the submission system before final acceptance, as unverified ORCID iDs will not appear in the published article. *Only*  the individual author can complete the verification step; PLOS staff *cannot*  verify ORCID iDs on behalf of authors.

We look forward to receiving your revised manuscript.

Kind regards,

Emmanuel Olamijuwon, Ph.D

Academic Editor

PLOS Global Public Health

**Journal Requirements:**

If the reviewer comments include a recommendation to cite specific previously published works, please review and evaluate these publications to determine whether they are relevant and should be cited. There is no requirement to cite these works unless the editor has indicated otherwise.

**Additional Editor Comments (if provided):**

Throughout the title, abstract, and introduction, the manuscript repeatedly characterises the intervention as "dance-integrated educational videos" and positions dance as the medium through which health education is delivered. However, based on the intervention description and accompanying materials, the educational information is conveyed through text overlays, while the dance sequences appear to be general, non-instructional performances that are not related to the educational content or reinforce the eye health messages. Please reconsider: (i) the terminology used to describe the intervention and ensure that the title, abstract, introduction, and conclusions accurately reflect what was actually tested. (ii) any conclusions regarding the educational effectiveness of dance and these should be substantially tempered, as the current study design does not isolate the contribution of the dance component.

Given the participants were recruited from clinics, how do the authors interpret the finding that “while 168 (38.4%) had never attended an eye clinic” on page 12, LN 248-251

The purpose of the chi-squared (χ²) tests reported in Table 1 is unclear. Please specify the comparison or hypothesis being tested.

On page 12, LN 252-255, the authors stated that “Among participants who reported being diagnosed with an eye condition, the most common diagnoses were myopia, presbyopia, astigmatism, and hyperopia (Figure 1)”. Could the authors clarify how the data presented in Figure 1 were obtained and categorised? Several of the reported responses (e.g., astigmatism, conjunctivitis, and ocular superinfection) are technical diagnostic terms. Please indicate whether participants generated these terms spontaneously in response to open-ended questions, selected them from predefined response options, or whether participants' descriptions were subsequently coded by the research team into diagnostic categories. Additional detail on this process would aid interpretation of the findings.

Page 12, LN 258-266 and Page 15, LN 308-324, it’s not clear which table or figure the results originate from. While the text interprets the statistical findings, there is no accompanying table or figure presenting the underlying data.

The intervention videos are currently provided via Google Drive links in the supplementary material. Could the authors consider depositing the videos in a permanent, citable repository (e.g., Zenodo, Figshare, OSF) to ensure that the intervention remains available to readers, facilitate reproducibility, and allow future researchers to evaluate and replicate the intervention?

[Note: HTML markup is below. Please do not edit.]

**Reviewers' comments:**

[NOTE: If reviewer comments were submitted as an attachment file, they will be attached to this email and accessible via the submission site. Please log into your account, locate the manuscript record, and check for the action link "View Attachments". If this link does not appear, there are no attachment files.]

**Figure Resubmissions:**

# Author comment: Author response to Decision Letter 4

**DOI:** 10.1371/journal.pgph.0005585.r008

13 Aug 2026

Attachment Submitted filename: Response to Reviewers comments_14082026.docx

# Editor report: Decision Letter 4

**DOI:** 10.1371/journal.pgph.0005585.r009

**Authors:** Emmanuel Olamijuwon

1 Sep 2026

PGPH-D-25-03403R4

Feasibility and Immediate Effects of a Multimodal, Dance-Integrated Educational Video Package on Eye Health Knowledge and Intentions in Nigeria: A Pre-Post Pilot Study

PLOS Global Public Health

Dear Dr. Osuagwu,

Thank you for submitting your manuscript to PLOS Global Public Health. After careful consideration, we feel that it has merit but does not fully meet PLOS Global Public Health’s publication criteria as it currently stands. Therefore, we invite you to submit a revised version of the manuscript that addresses the points raised during the review process.

Please submit your revised manuscript by Oct 01 2026 11:59PM. If you will need more time than this to complete your revisions, please reply to this message or contact the journal office at globalpubhealth@plos.org. When you're ready to submit your revision, log on to https://www.editorialmanager.com/pgph/ and select the 'Submissions Needing Revision' folder to locate your manuscript file.

Please include the following items when submitting your revised manuscript:

- A letter that responds to each point raised by the editor and reviewer(s). You should upload this letter as a separate file labeled 'Response to Reviewers'.
- A marked-up copy of your manuscript that highlights changes made to the original version. You should upload this as a separate file labeled 'Revised Manuscript with Track Changes'.
- An unmarked version of your revised paper without tracked changes. You should upload this as a separate file labeled 'Manuscript'.

Guidelines for resubmitting your figure files are available below the reviewer comments at the end of this letter.

As the corresponding author, your ORCID iD is verified in the submission system and will appear in the published article. PLOS supports the use of ORCID, and we encourage all coauthors to register for an ORCID iD and use it as well. Please encourage your coauthors to verify their ORCID iD within the submission system before final acceptance, as unverified ORCID iDs will not appear in the published article. *Only*  the individual author can complete the verification step; PLOS staff *cannot*  verify ORCID iDs on behalf of authors.

We look forward to receiving your revised manuscript.

Kind regards,

Emmanuel Olamijuwon, Ph.D

Academic Editor

PLOS Global Public Health

**Journal Requirements:**

If the reviewer comments include a recommendation to cite specific previously published works, please review and evaluate these publications to determine whether they are relevant and should be cited. There is no requirement to cite these works unless the editor has indicated otherwise.

Please review your reference list to ensure that it is complete and correct. If you have cited papers that have been retracted, please include the rationale for doing so in the manuscript text, or remove these references and replace them with relevant current references. Any changes to the reference list should be mentioned in the rebuttal letter that accompanies your revised manuscript. If you need to cite a retracted article, indicate the article’s retracted status in the References list and also include a citation and full reference for the retraction notice.

**Additional Editor Comments (if provided):**

- The manuscript would still benefit from further clarification on the rationale for the dance-integrated videos. The manuscript states that “These traditional modalities are deeply rooted in the 113 country’s communal and oral traditions, providing a historically proven framework for disseminating critical health information in ways that resonate with diverse audiences, regardless of literacy levels”, yet participants in this study needed English literacy to understand the messages being conveyed; so this particular justification doesn't quite hold here. Relatedly, the pathways linking dance to outcomes such as cardiovascular health and self-efficacy are well established as cited, but the mechanism for eye health education is less clear. It would help to justify more directly why dance and allied performing arts are suited to eye health promotion. One plausible pathway is engagement since long texts can be easy to disengage from, whereas interactive dance with text overlays may capture attention and encourage fuller reading of the information provided. Some of this is already touched on in the discussion — "Dance, as a universally accessible form of art, has been leveraged in health campaigns to attract attention, enhance recall, and foster community-level diffusion [32–36, 45, 46]", and could be brought forward and emphasised in the introduction.
- The phrase "a pre–post interventional pilot intervention study" repeats the idea of intervention twice. I'd suggest simplifying to "a pre–post pilot intervention study"
- The 18–44 age band is quite broad to be grouped together as "young adults." Could the authors provide a rationale for this grouping, or, where sample size allows, disaggregate it?
- In Tables 3 and 5, the chi-square analyses have sparse expected cell counts (4 of 15 cells < 5), which can make the chi-square approximation unreliable. It would strengthen the analysis to confirm these results with an exact R×C test (e.g., Fisher–Freeman–Halton) or a Monte Carlo p-value.
- Given the single-group pre–post design and the immediate post-intervention assessment, I'd suggest moderating the causal language throughout. The findings indeed demonstrate an immediate change in knowledge following the intervention, but the design cannot exclude alternative explanations or establish longer-term effects. For example, "Effect of the number of videos watched on survey items" might be reframed as "Association between the number of videos watched and survey responses," with similar softening of causal phrasing elsewhere.
- Thank you for clarifying that the diagnostic terms in Figure 1 reflect participants' selections from the predefined categories in the questionnaire. Several of these (e.g., astigmatism, conjunctivitis, ocular superinfection) are fairly technical, which raises a question about whether participants, particularly those with primary or no formal education, could reliably identify their own diagnosis. Could the authors clarify how these terms were presented and explained, and whether any steps were taken to help participants understand the categories well enough to make an accurate selection? or whether there is a potential for under-reporting especially among less-educated participants?

[Note: HTML markup is below. Please do not edit.]

**Reviewers' comments:**

[NOTE: If reviewer comments were submitted as an attachment file, they will be attached to this email and accessible via the submission site. Please log into your account, locate the manuscript record, and check for the action link "View Attachments". If this link does not appear, there are no attachment files.]

**Figure Resubmissions:**

# Author comment: Author response to Decision Letter 5

**DOI:** 10.1371/journal.pgph.0005585.r010

17 Sep 2026

Attachment Submitted filename: Response to Editor Comments.170926.docx

# Editor report: Decision Letter 5

**DOI:** 10.1371/journal.pgph.0005585.r011

**Authors:** Emmanuel Olamijuwon

21 Sep 2026

Feasibility and Immediate Effects of a Multimodal, Dance-Integrated Educational Video Package on Eye Health Knowledge and Intentions in Nigeria: A Pre-Post Pilot Study

PGPH-D-25-03403R5

Dear Dr Osuagwu,

We are pleased to inform you that your manuscript 'Feasibility and Immediate Effects of a Multimodal, Dance-Integrated Educational Video Package on Eye Health Knowledge and Intentions in Nigeria: A Pre-Post Pilot Study' has been provisionally accepted for publication in PLOS Global Public Health.

Before your manuscript can be formally accepted you will need to complete some formatting changes, which you will receive in a follow up email. A member of our team will be in touch with a set of requests.

Please note that your manuscript will not be scheduled for publication until you have made the required changes, so a swift response is appreciated.

IMPORTANT: The editorial review process is now complete. PLOS will only permit corrections to spelling, formatting or significant scientific errors from this point onwards. Requests for major changes, or any which affect the scientific understanding of your work, will cause delays to the publication date of your manuscript.

If your institution or institutions have a press office, please notify them about your upcoming paper to help maximize its impact. If they'll be preparing press materials, please inform our press team as soon as possible -- no later than 48 hours after receiving the formal acceptance. Your manuscript will remain under strict press embargo until 2 pm Eastern Time on the date of publication. For more information, please contact globalpubhealth@plos.org.

Thank you again for supporting Open Access publishing; we are looking forward to publishing your work in PLOS Global Public Health.

Best regards,

Emmanuel Olamijuwon, Ph.D

Academic Editor

PLOS Global Public Health

***********************************************************

Reviewer Comments (if any, and for reference):
